# Valepotriates From the Roots and Rhizomes of *Valeriana jatamansi* Jones as Novel N-Type Calcium Channel Antagonists

**DOI:** 10.3389/fphar.2018.00885

**Published:** 2018-08-13

**Authors:** Fa-Wu Dong, He-Hai Jiang, Liu Yang, Ye Gong, Cheng-Ting Zi, Dan Yang, Chen-Jun Ye, Huan Li, Jian Yang, Yin Nian, Jun Zhou, Jiang-Miao Hu

**Affiliations:** ^1^State Key Laboratory of Phytochemistry and Plant Resources in West China, Yunnan Key Laboratory of Natural Medicinal Chemistry, Kunming Institute of Botany, Chinese Academy of Sciences, Kunming, China; ^2^Faculty of Pharmacy, Yunnan University of Traditional Chinese Medicine, Kunming, China; ^3^Key Laboratory of Bioactive Peptides of Yunnan Province, Key Laboratory of Animal Models and Human Disease Mechanisms of Chinese Academy of Sciences, Ion Channel Research and Drug Development Center, Kunming Institute of Zoology, Chinese Academy of Sciences, Kunming, China; ^4^Faculty of Life Science and Technology, Kunming University of Science and Technology, Kunming, China; ^5^Department of Biological Sciences, Columbia University, New York, NY, United States

**Keywords:** *Valeriana jatamansi*, iridoid, abdominal distention and pain, N-type voltage-gated calcium channels, Ca_v_2.2

## Abstract

The roots and rhizomes of *Valeriana jatamansi* have long been used as folk medicine in Asia and usually named as “Zhizhuxiang” in Chinese for the treatment of abdominal distention and pain. However, its active ingredients and molecular targets for treatment of abdominal pain remain unrevealed. Inhibitors of Ca_v_2.2 N-type voltage-gated calcium channels (VGCCs) are actively sought after for their potential in treating pain, especially chronic pain. As far as we know, the method used for seeking analgesic active ingredient from plant material has rarely been reported. The analgesic potentials of the EtOH extract (0.01 mg/ml) of the roots and rhizomes of *V. jatamansi* and its EtOAc, *n*-BuOH and H_2_O soluble parts (0.01 mg/ml, respectively) were tested herein on Ca_v_2.2, using whole-oocyte recordings *in vitro* by tow-electrode voltage clamp. The results indicated that the EtOAc-soluble part exhibited the most potent inhibition of Ca_v_2.2 peak current (20 mv). The EtOAc-soluble part was then subjected to silica gel column chromatography (CC) and giving 9 fractions. Phytochemical studies were carried out by repeated CC and extensive spectroscopic analyses after the fraction (0.01 mg/ml) was identified to be active and got seventeen compounds (**1**–**17**). All isolates were then sent for further bioactive verification (**1** and **3** at concentration of 10 μM, others at 30 μM). In addition, the selectivity of the active compounds **1** and **3** were tested on various ion channels including Ca_v_1.2, Ca_v_2.1 and Ca_v_3.1 VGCCs and Kv1.2, Kv2.1, Kv3.1 and BK potassium channels. The results indicated that compound **1** and **3** (an abundant compound) inhibited Ca_v_2.2 with an EC_50_ of 3.3 and 4.8 μM, respectively, and had weaker or no effect on Ca_v_1.2, Ca_v_2.1 and Ca_v_3.1 VGCCs and Kv1.2, Kv2.1, Kv3.1 and BK potassium channels. Compounds **1** and **3** appear to act as allosteric modulators rather than pore blockers of Ca_v_2.2, which may play crucial role in attenuating nociception. The results of present research indicated that the ethnopharmacological utilization of *V. jatamansi* for relieving the abdominal distention and pain may mediate through Ca_v_2.2 channel. Our work is the first demonstration of inhibition of Ca_v_2.2 by iridoids, which may provide a fresh source for finding new analgesics.

## Introduction

N-type calcium channels (Ca_v_2.2) are highly distributed at nerve terminals and on cell bodies of dorsal root ganglia (DRG) neurons, where they regulate the release of pain-related neuropeptides. Pharmacology and genetic studies showed that Ca_v_2.2 inhibiting activity was an effective way to manage or alleviate symptoms of inflammatory, chronic and neuropathic pain (Kerr et al., 1988; Gohil et al., 1994; Saegusa et al., 2001, 2002; McGivern and McDonough, 2004; McGivern, 2006; Altier et al., 2007; Trang et al., 2015). Ca_v_2.2 antagonists have therefore long been considered as potential analgesics (Hazelhoff et al., 1982; Mathela et al., 2005; La et al., 2008; Yamamoto and Takahara, 2009; Pajouhesh et al., 2010; Schmidtkco et al., 2010; Swensen et al., 2014). Indeed, a synthetic form of ω-conotoxin MVIIA, a peptide toxin isolated from the marine cone snail, genus *Conus*, that potently inhibits Ca_v_2.2, is in clinical use (marked as ziconotide) to treat severe and chronic pain (Schmidtkco et al., 2010). However, ziconotide can only be delivered intrathecally and even so has numerous serious adverse effects. Therefore, it is clearly desirable for development of new Ca_v_2.2 antagonists.

*Valeriana jatamansi* Jones distributed widely in China and some other Asian countries (Mathela et al., 2005). The roots and rhizomes of this plant is a well-known herbal medicine “Zhizhuxiang” in the Chinese Pharmacopoeia for treating abdominal pain (Editorial Board of Chinese Pharmacopoeia, 2015). Several clinically used medicines for treating abdominal pain in the Chinese market, such as “Xiangguo Jianxiao Pian,” are prepared by Zhizhuxiang as a main component. However, the chemical basis and molecular targets of *V. jatamansi* Jones for the treatment of abdominal pain remain unrevealed.

**FIGURE 1:**
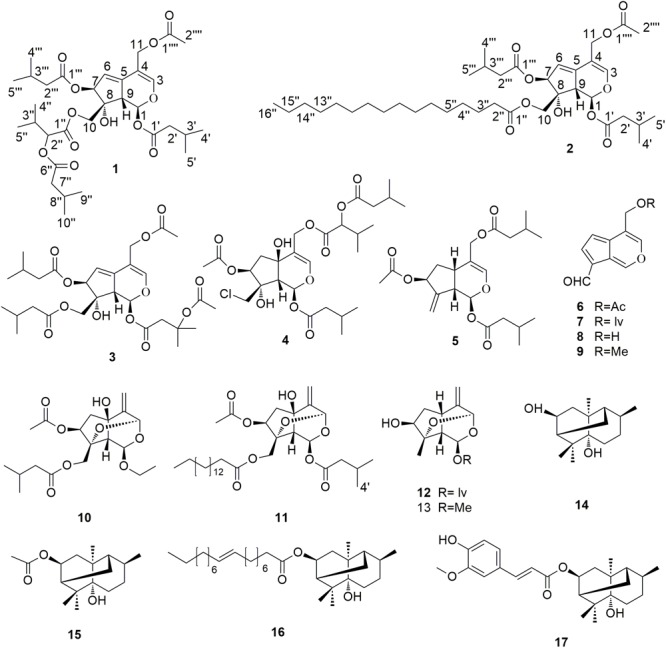
Chemical structures of compounds **1–17**.

Inspired by the analgesic effect of *V. jatamansi* Jones, bioassay-guided isolation and characterization of the active constituents of the plant that target Ca_v_2.2 were carried out herein. Initially, the ethanol extract and the EtOAc, *n*-BuOH and H_2_O fractions of the plant were tested for their inhibition of Ca_v_2.2. Subsequent function-guided phytochemical studies on the active EtOAc part resulted in the isolation and identification of thirteen iridoids (**1**–**13**) and four sesquiterpenes (**14**–**17**) (**Figure 1**), including two new valepotriates, namely jatamanvaltrate T and U (**1**–**2**). The inhibitory activities on Ca_v_2.2 of all the compounds were tested. Among them, the new compound **1** and the main constituent **3** (8.45 g out of 32.5 kg of plant material) exhibited significant inhibitory effects on Ca_v_2.2 with EC_50_ values of 3.3 and 4.8 μM, respectively. In addition, compound **1** and **3** showed noticeable selectivity over Ca_v_1.2, Ca_v_2.1 and Ca_v_3.1 VGCCs and Kv1.2, Kv2.1, Kv3.1 and BK potassium channels.

## Materials and Methods

### General Experimental Procedures

Optical rotations were measured with a JASCO model 1020 polarimeter (Horiba, Tokyo, Japan). UV spectra were recorded on a Shimadzu UV-2401PC spectrophotometer (Shimadzu, Kyoto, Japan) and IR (KBr) spectra were obtained using a Bruker Tenor 27 FT-IR spectrophotometer (Bruker Optics GmbH, Ettlingen, Germany). ESIMS and HRESIMS were performed on an Agilent G6230 TOF MS (Agilent Technologies, Palo Alto, CA, United States) or an API QSTAR time-of-flight spectrometer (AB-MDS Sciex, Concord, ON, Canada) or a VG Autospec-3000 spectrometer (VG, Manchester, United Kingdom). 1D and 2D NMR spectra were recorded on Bruker AM-400 and DRX-500 spectrometer (Bruker, Bremerhaven, Germany) with TMS as the internal standard. Chemical shifts (δ) are expressed in ppm with reference to the solvent signals. MPLC was run on a Dr-Flash-S MPLC system (Lisui, Suzhou, China). Silica gel (200-300 mesh) for column chromatography (CC) and TLC was obtained from Qingdao Marine Chemical Factory, Qingdao, China. Sephadex LH-20 was purchased from Amersham Biosciences, Sweden. RP-C18 gel (40–63 μm, Merck, Darmstadt, Germany) and MCI gel (75–150 μm, Mitsubishi Chemical Corporation, Japan). Fractions were monitored by TLC (GF254, Qingdao Haiyang, Qingdao, China), and spots were visualized by sprayed with 5% sulfuric acid in EtOH, followed by heating.

### Plant Material

The roots and rhizomes of *V. jatamansi* were collected at Kunming, Yunnan Province, People’s Republic of China, in July 2012, and identified by Prof. En-De Liu, and a voucher specimen (KUN NO. 0864803), has been deposited in State Key Laboratory of Phytochemistry and Plant Resource in West China, Kunming Institute of Botany, The Chinese Academy of Sciences, Kunming, China.

### The Preparation of Crude Extract and Fractions

The air-dried roots (450 g) of *V. jatamansi* were powdered and extracted with 95% ethanol at room temperature (3L × four times, each time 24 h) with occasional shaking. The extracted was filtered and concentrated with a rotary evaporator at 45–50°C, giving 75.5 g residues (The yield is approximate 16.8%). The ethanol extract (43.62 g) was suspended in distilled (600 ml) water and partitioned successively with EtOAc and *n*-BuOH (five times, each time 0.5 h), then evaporated on rotary evaporator to afford EtOAc-soluble part (12.73 g) and *n*-BuOH layer (5.38 g). The H_2_O-soluble part was passed through a macroporous absorbent resin (D-101) with H_2_O and ethanol, then concentrated under reduced pressure to obtain H_2_O layer (0.51 g). The EtOAc part was subjected to silica gel column chromatography eluted with a gradient of petroleum ether/acetone (1:0-0:1, v/v) to afford 9 fractions (Fr.1–Fr.9).

### Extraction and Isolation

Based on the observed effect with the EtOAc portion on Ca_v_2.2, further extraction and isolation of the EtOAc portion were carried out herein.

The air-dried, powdered roots of *V. jatamansi* (32.50 kg) were extracted three times with 95% EtOH (3 × 60 L, each 28 h) at room temperature. The combined EtOH extracts were concentrated in vacuum to afford a crude residue (3.65 kg), which was then suspended in water (4.0 L) and partitioned with EtOAc (4.0 L × 5). Then got the EtOAc part (1.72 kg) and chromatographed on a silica gel column chromatography (CC, 200–300 mesh), eluting with a gradient of petroleum ether-acetone (1:0-0:1) to give nine fractions (Fr.1–Fr.9). Fr.1 (192.4 g) was resubmitted to silica gel CC (petroleum ether-acetone gradient, 1:0-10:1) and then purified by Sephadex LH-20 column (MeOH) to yield compound **5** (94.0 mg) and **17** (17 mg). Similarly, **8** (53 mg) and **6** (135 mg) were got from Fr.2 (41.10 g). Fr.3 (55.50 g) was decolorized on a MCI gel (MeOH-H_2_O gradient, 60:40-95:5), then subjected to MPLC (RP-18 gel, MeOH-H_2_O gradient, 40:60-90:10) and separated by Sephadex LH-20 column (MeOH) to give **7** (246 mg) and **10** (34 mg). The bioactive Fr.4 (100.30 g) was chromatographed on silica gel CC (200–300 mesh) (petroleum ether-acetone, 50:1-1:1) to yield 4 fractions (Fr.4.1–Fr.4.4). Fraction 4.2 (28.3 g) was applied to a MCI gel (MeOH-H_2_O gradient, 60:40-85:15), then by repeated silica gel CC (petroleum ether-acetone, 20:1-5:1) to obtain **14** (70 mg) and **15** (1.65 g), then further by RP-C18 (MeOH-H_2_O gradient, 50:50-85:15) and followed by Sephadex LH-20 (MeOH) to give **1** (148 mg) and **9** (95 mg). Fr.4.3 (22.017 g) was successively separated by MPLC (MeOH-H_2_O gradient, 30:60-50:50) and repeated silica gel CC (petroleum ether-EtOAc, 10:1-5:1), purified by Sephadex LH-20 (MeOH) to obtain **2** (81 mg) and **11** (5 mg). The bioactive Fr.6 (193.70 g) was passed through a silica gel CC and eluted with a gradient of petroleum ether-acetone (20:1-0:1) to yield 11 fractions (Fr.6.1–Fr.6.11). Fr.6.8 (70.0 g) was subjected to silica gel CC (petroleum ether-acetone gradient, 10:1-0:1) to afford 8 sub-fractions (Fr.6.8.1–Fr.6.8.8). Fr.6.8.5 (33.64 g) was repeatedly subjected to silica gel CC (petroleum ether-acetone gradient, 10:1-0:1), then purified by Sephadex LH-20 column (MeOH) to give **3** (8.45 g) and **4** (50 mg) and **16** (60 mg). Fr.6.9 (73.5 g) was treated as Fr.6.8.5 and get compounds **12** (20 mg) and **13** (51 mg).

### Physical and Spectroscopic Data of New Compounds

#### Jatamanvaltrate T (1)

Colorless oil, ${\left[a\right]}_{D}^{23}$ = +152.60 (*c* 0.26, MeOH, **Supplementary Figure S12**). UV (MeOH) λ_max_ (log 𝜀_max_): 255 (5.22) nm (**Supplementary Figure S11**); IR (KBr) ν_max_ (cm^-1^): 3441, 2963, 2875, 1742, 1640, 1467, 1371, 1294, 1250, 1187, 1166, 1101, 1027, 963 cm^-1^; ^1^H NMR and ^13^C NMR data, see **Table 1**; positive ESIMS m/z 647 [M + Na]^+^; HRESIMS m/z 647.3040 [M + Na]^+^ (calculated for C_32_H_48_O_12_Na, 647.3043).

**Table 1:** ^1^H NMR (400 MHz) and ^13^C NMR (100 MHz) data of compounds 1–2 in CD_3_OD.

	1		2	
Position	δ_H_	δ_C_	δ_H_	δ_C_
1	6.11, d (10.0)	94.0, CH	6.12, d (10.1)	94.0, CH
3	6.78, s	149.5, CH	6.77, s	149.4, CH
4		110.4, C		110.4, C
5		140.6, C		140.7, C
6	5.76, dd (2.6, 2.5)	118.6, CH	5.74, dd (2.7, 2.6)	118.4, CH
7	5.48, d (2.7)	84.5, CH	5.49, d (2.7)	84.5, CH
8		80.6, C		80.6, C

*The ^*1*^H NMR data of the substituents at C-10 of****1****(α-(isovaleroxy)isovaleroxy) group): 2.27 (2H, m, H-7″), 2.11 (1H, m, H-8″), and 0.98 (6H, d, J = 6.4 Hz, H-9″, 10″); and of****2****(palmitoyl):1.28 (20H, m, H-6″∼15″) and 0.88 (3H, t, J = 5.6 Hz, H-16″). The ^*13*^C NMR data of the substituents at C-10 of****1****(α-(isovaleroxy)isovaleroxy) group): δ_*C*_ 174.0 (C, C-6″), 43.8, (CH_*2*_, C-7″), 26.9, (CH, C-8″) and 22.7 (CH_*3*_, C-9″, 10″); and of****2****(palmitoyl): δ_*C*_ 30.2-30.7 (CH_*2*_, C-6″-13″), 33.0 (CH_*2*_, C-14″), 23.5 (CH_*2*_, C-15″) and 14.1 (CH_*3*_, C-16″).*

#### Jatamanvaltrate U (2)

White amorphous powder, ${\left[a\right]}_{D}^{23}$ = +131.61 (*c* 0.45, MeOH) (**Supplementary Figure S22**). UV (MeOH) λ_max_ (log 𝜀_max_):: 255 (4.59) nm (**Supplementary Figure S21**); IR (KBr) ν_max_ (cm^-1^): 3444, 2923, 1733, 1642, 1467, 1381, 1296, 1260, 1183, 1154, 1101, 1082, 1016, 987 cm^-1^; ^1^H NMR and ^13^C NMR data, see **Table 1**; positive ESIMS m/z 701 [M + Na]^+^; HRESIMS m/z 701.4234 [M + Na]^+^ (calculated for C_38_H_62_O_10_Na, 701.4241).

### Bioassay

#### Oocyte Preparation and Expression

Oocytes were obtained from adult *Xenopus* by digesting its ovarian lobes with collagenase A (sigma) for 2–3 h under 180 rpm shaking in OR2 [MgCl_2_ (1 mM), NaCl (82.4 mM), KCl (2.5 mM), HEPES (5 mM) and NaOH (pH 7.6)]. The best Stages V–VI oocytes were selected, injected with cRNA (50–100 ng), and then incubated at 18°C for 3–6 days depending on cRNA expression in ND96 [CaCl_2_ (1.8 mM), MgCl_2_ (1 mM), NaCl (96 mM), KCl (2.5 mM), HEPES (5 mM), streptomycin (100 μg/mL), penicillin (100 units/mL) and NaOH (pH 7.6)].

#### HEK 293T Cell Culture and Expression

Human embryonic kidney (HEK) 293T cells were grown in DMEM (HyClone) plus 10% newborn calf serum (Gibco) and streptomycin (0.1 mg/ml)/penicillin (100 U/ml) (Biological Industries). The cells were transiently transfected with pCDNA3.1-α_2_δ, pCDNA3.1-β_3_, pCDNA3.1-EGFP and pCDNA3.1-N type plasmids together using LipoD293^TM^ (SignaGen Laboratories) and recorded in 48 h.

#### Electrophysiology

All experiments were performed at 20–22°C. Whole-oocyte recordings by performed with two-electrode voltage clamp (TEVC). Electrodes were filled with KCl (3 mM) and had resistances of 0.3–1 MΩ. The bath solution used to record calcium channel currents contained KCl (2 mM), BaCl_2_ (1.8 mM), NaOH (50 mM), Ba(OH)_2_ (40 mM) and HEPES (5 mM). pH 7.4 was adjusted with methanesulfonic acid and the solution was filtered. L-type (Ca_v_1.2), N-type (Ca_v_2.2), and P/Q-type (Ca_v_2.1) calcium channel currents were evoked from a holding potential of -80 mV by 50-ms depolarizations ranging from -30 to 70 mV in 10-mV increments at 3-s intervals. Currents through T-type (Ca_v_3.1) calcium channels were elicited by voltage pulses (50 ms) from -50 to 60 mV with a holding potential of -80 mV in 10-mV increments at 3-s intervals. Kv1.1 currents were recorded from a holding potential of -80 mV by 200-ms depolarizations ranging from -60 to 60 mV in 10-mV increments at 15-s intervals. Kv2.1 and Kv3.1 currents were obtained from a holding potential of -80 mV by 200-ms depolarizations ranging from -40 to 60 mV in 10-mV increments at 15-s intervals. BK channel currents were obtained from a holding potential 0f -50 mV by 60-ms depolarization ranging from 0 to 100 mV in 10-mV increments at 3-s intervals. All the currents were sampled and filtered, respectively.

In whole-cell recordings of HEK 293T cells, pipettes were fabricated from borosilicate glass (World Precision Instruments) using a micropipette puller (P-1000, Sutter Instrument), and were fire-polished to resistances of ∼3 MW. Whole-cell currents were elicited by 20-ms voltage steps from -60 to 80 mV with 10-mV increments, with a holding potential of -80 mV. Currents were amplified by Axopatch 200B and digitized by Digidata 1440A (Molecular Devices). Currents were low-pass filtered at 2 kHz and sampled at 10 kHz. The extracellular solutions contained (in mM) 5 CsCl, 10 BaCl_2_, 140 TEA.Cl, 10 Glucose and 10 HEPES. pH was adjusted to 7.4 with CsOH. The intracellular solutions contained (in mM) 4 MgCl_2_, 140 CsCl, 10 EGTA and 10 HEPES (pH 7.4 adjusted with CsOH).

The effect on Ca_v_2.2 of the ethanol extract (0.01 mg/ml) and the EtOAc, *n*-BuOH and H_2_O soluble parts (0.01 mg/ml) of the EtOH extract was tested by TEVC of *Xenopus* oocytes (**Supplementary Figure S23**). The EtOAc-soluble part (**Supplementary Figure S24**), which exhibited a stronger inhibitory activity, was subjected to silica gel column chromatography to produce 9 fractions. All of the fractions (0.01 mg/ml), together with the isolates (**1**–**17**) from the fractions, were tested for their inhibitory effects on Ca_v_2.2, with a concentration of 10 μM (**1** and **3**) or 30 μM (**2**, and **4**–**17**). Compounds **1** and **3** were further tested for their activities on Ca_v_1.2, Ca_v_2.1 and Ca_v_3.1 VGCCs (10 μM) and on Kv1.2, Kv2.1, Kv3.1 and BK potassium channels (30 μM) (**Supplementary Figure S25**).

In a positive control experiment, the effect on Ca_v_2.2 of 100 μM CdCl_2_ and 0.2–1.0 μM ω-conotoxin MVIIA was tested by TEVC in *Xenopus* oocytes and by whole-cell recording in HEK 293T cells.

#### Data Analysis and Statistics

Data acquisition and analysis of the whole-oocyte recordings were carried out by using pClamp 10 (Molecular Devices Corporation, San Jose, CA, United States). Data fitting and statistical analyses were performed by PRISM 5.0 (GraphPad Software Inc., San Diego, CA, United States). EC_50_ values and Hill slopes were determined by fitting the data points to a sigmoidal dose-response equation (*Y* = Min + (Max-Min)/(1+10ˆ((LogEC50-*X*)^∗^*n*))), where *Y* is % Inhibition, *X* is the concentration of the compounds, Min is minimal inhibition, Max is maximum inhibition, and *n* is the Hill coefficient. All data were presented as mean ± SEM, and statistical analysis was performed using Student’s *t*-test. *P*-values of < 0.05 were considered as significant, and levels of significance were marked by asterisks (^∗∗^*P* < 0.01, ^∗^*P* < 0.05).

## Results and Discussion

### Structural Elucidation of Isolated Compounds

**FIGURE 2:**
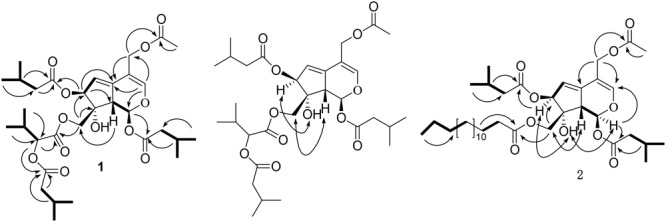
Key ^1^H-^1^H COSY (**–**), HMBC (→), and ROESY (↔) correlations of **1** and **2**.

Jatamanvaltrate T (**1**) was obtained as colorless oil with the molecular formula of C_32_H_48_O_12_ assigned by positive HRESIMS (**Supplementary Figure S1**) at m/z 647.3040 [M + Na]^+^ (calcd for C_32_H_48_O_12_Na, 647.3043), indicating nine degrees of unsaturation. The IR absorption (**Supplementary Figure S10**) bands at 3441, 1742, and 1640 cm^-1^ revealed the presence of hydroxy, ester carbonyl, and double bond groups, respectively. The ^1^H NMR and ^13^C-NMR spectroscopic data (**Table 1** and **Supplementary Figures S2–S4**) displayed signals for two trisubstituted olefinic bonds [δ_H_ 6.78 (s, H-3); δ_C_ 149.5 (d, C-3) and 110.4 (s, C-4); δ_H_ 5.76 (dd, *J* = 2.6, 2.5 Hz, H-6); δ_C_ 118.6 (d, C-6) and 140.6 (s, C-5)], a hemiketal methine [δ_H_ 6.11 (d, *J* = 10.0 Hz), H-1); δ_C_ 94.0 (d, C-1)], and two oxymethylenes [δ_H_ 4.39, 4.35 (each d, *J* = 11.3 Hz, H-10), 4.77 (d, *J* = 11.4 Hz, H-11a) and 4.65 (d, *J* = 12.3 Hz, H-11b); δ_C_ 67.5 (t, C-10) and 62.0 (t, C-11)]. These data, together with the resonances of five ester carbonyls at δ_C_ 171.0, 172.1, 172.6, 173.8, and 174.0, led to the assumption that compound **1** is a valtrate hydrin-type iridoid (Demirezer et al., 1999; Tang et al., 2002; Wang et al., 2008, 2014; Lin et al. 2010b, 2013). It could be seen that the structure of **1** was similar to those of 10-acetoxy-1-acevaltrate hydrin from the ^1^H- and ^13^C-NMR data of **1** (Tang et al., 2002), except for the presence of an isovaleroxy residue [δ_C_ 172.1 (s, C-1′), 44.0 (t, C-2′), 26.8 (d, C-3′), 22.6 (q, C-4′, C-5′) and an α-(isovaleroxy)isovaleroxy group [δ_C_ 171.0 (s, C-1″), 77.8 (d, C-2″), 31.1 (d, C-3″), 19.3 (q, C-4″), 17.5 (q, C-5″), 174.0 (s, C-6″), 43.8, (t, C-7″), 26.9, (d, C-8″), and 22.7 (q, C-9″, 10″)] in **1**. The substituent structure of **1** with two isovaleroxy groups located at C-1 and C-7, respectively, was confirmed by the HMBC spectra (**Supplementary Figures S5–S7**) as following: the correlations (**Figure 2**) from H-1 (δ_H_ 6.11, d, *J* = 10.0 Hz), H-2′ (δ_H_ 2.32, m) and H-3′ (δ_H_ 2.04, m) to C-1′ (δ_C_ 172.1, s); from H-7 (δ_H_ 5.48, d, *J* = 2.7 Hz), H-2′″ (δ_H_ 2.28, m) and H-3′″ (δ_H_ 2.15, m) to C-1′″ (δ_C_ 173.8, s). The connection of the α-(isovaleroxy)isovaleroxy functionality to C-10 was fully determined on the basis of the correlations from H-10 (δ_H_ 4.39, 4.35, each d, *J* = 11.3 Hz), H-2″ (δ_H_ 4.78, d, *J* = 4.4 Hz) and H-3″ (δ_H_ 2.26, m) to C-1″ (δ_C_ 171.0, s), together with connections from H-2″ (δ_H_ 4.78, d, *J* = 4.4 Hz), H-7″ (δ_H_ 2.27, m) and H-8″ (δ_H_ 2.11, m) to C-6″ (δ_C_ 174.0, s). The correlations from H-11a (δ_H_ 4.77, d, *J* = 11.4 Hz), H-11b (δ_H_ 4.65, d, *J* = 12.3 Hz) and H-2″″ (δ_H_ 2.00, s) to C-1″″ (δ_C_ 172.6, s) determined the linkage of the AcO residue to C-11.

The relative configuration of **1** was further established by ROESY experiment (**Figure 2** and **Supplementary Figures S8, S9**) and comparison the spectroscopic data with those reported valepotriates (Demirezer et al., 1999; Tang et al., 2002; Wang et al., 2008, 2014; Lin et al., 2010b, 2013). Generally, naturally occurring iridoids display α-orientation for H-1 and β-orientation for H-9. In the ROESY spectrum, the correlations of H-9/H-10, OH-8/H-7, not of H-7/H-9 and H-7/H-10 implied that H-9 and H-10 were β-oriented, and H-7 and OH-8 were α-oriented. Comparing the NMR data of **1** with iridoids reported in the literature, the configurations of H-1, H-7, OH-8, H-9, and H-10 were consistent with those valepotriates (Demirezer et al., 1999; Wang et al., 2008, 2014; Lin et al., 2010b, 2013). Thus, **1** was assigned as (1*S*, 7*S*, 8*R*, 9*S*)-11-acetoxy-10-[a-(isovaleroxy)isovaleroxy]-1, 7-diisovaleroxyvaltrate hydrin, named as jatamanvaltrate T.

Jatamanvaltrate U (**2**) was get as an amorphous white powder, possessed the molecular formula of C_38_H_62_O_10_ by positive-ion HRESIMS at *m*/*z* 701.4234 [M + Na]^+^ (calcd 701.4241) (**Supplementary Figure S13**). Its IR spectrum (**Supplementary Figure S20**) revealed the presence of hydroxy, ester carbonyl and double bond groups from absorption bands at 3444, 1733, and 1642 cm^-1^. Detailed analysis of the ^1^H, ^13^C NMR and DEPT data of **2** (**Table 1**) (**Supplementary Figures S14, S15**) revealed that the structure of **2** was similar to those of **1** except for the presence of a palmitoyl group [δ_H_ 2.32 (2H, t, *J* = 2.4 Hz, H-2″), 1.59 (2H, m, H-3″), 1.28 (each 2H, each m, H-4″∼15″) and 0.88 (3H, t, *J* = 5.6 Hz, H-16″); δ_C_ 175.0 (s, C-1″), 34.5 (t, C-2″), 25.9 (t, C-3″), 30.2-30.7 (t, C-4″-13″), 33.0 (t, C-14″), 23.5 (t, C-15″), and 14.1 (q, C-16″)] in **2** rather than an α-(isovaleroxy)isovaleroxy in **1**. The HMBC correlations (**Figure 2**) of H-10 (δ_H_ 4.31, brs), H-2″ (δ_H_ 2.32, t, *J* = 2.4 Hz) and H-3″ (δ_H_ 1.59, m) with C-1″ (δ_C_ 175.0, s), of H-11a (δ_H_ 4.76, d, *J* = 12.3 Hz), H-11b (δ_H_ 4.65, d, *J* = 12.3 Hz), and H-2″″ (δ_H_ 2.00, s) with C-1″″ (δ_C_ 172.6, s) indicated the palmitoyl and acetoxyl residue were located at C-10 and C-11 of **2**, respectively. Furthermore, the positions of the other two isovaleroxy groups at C-1 and C-7 were also confirmed based on the HMBC correlations (**Supplementary Figures S16–S18**).

By comparison of the ROESY correlations (**Supplementary Figure S19**) with that of **1**, the relative configuration of **2** (**Figure 2**) was demonstrated to be identical to that of **1**. All of the key ROESY correlations of H-9/H-10, OH-8/H-7 supporting the structure of **1** were also observed in **2**, suggesting that the H-9 and H-10 were β-oriented and H-7 and OH-8 were α-oriented. Therefore, **2** was established as (1*S*, 7*S*, 8*R*, 9*S*)-11-acetoxy-1,7-di isovaleroxy-10-palmitoyl caltrate hydrin, named as jatamanvaltrate U.

The known compounds, valtrate hydrin B8 (**3**) (Demirezer et al., 1999), volvaltrate B (**4**) (Lin et al., 2010b), deoxido-didrovaltrate (**5**) (Bounthanh et al., 1983), baldrinal (**6**) (Denee et al., 1979), homobaldrinal (**7**) (Denee et al., 1979), desacylbaldrinal (**8**) (Georg and Michael, 1979), 11-methoxyviburtinal (**9**) (Chen et al., 2005), jatamanvaltrate R (**10**) (Dong et al., 2015), jatamanvaltrate S (**11**) (Dong et al., 2015) rupesin E (**12**) (Lin et al., 2010a), (1S, 3R, 5S, 7S, 8S, 9S)-3, 8-epoxy-7-hydroxy-1-methoxy-4, 11-dihydronepetane (**13**) (Thies, 1970), valeriananoid B (**14**) (Ming et al., 1997), valeriananoid C (**15**) (Ming et al., 1997), valeriananoid D (**16**) (Dong et al., 2015), and valeriananoid E (**17**) (Dong et al., 2015) were identified by analysis of their spectroscopic data and comparison with the literature values. This is the first isolation of iridoids with fatty acid esters (**2**) from the family of Valerianaceae.

### Functional Characterization of Isolated Compounds

**FIGURE 3:**
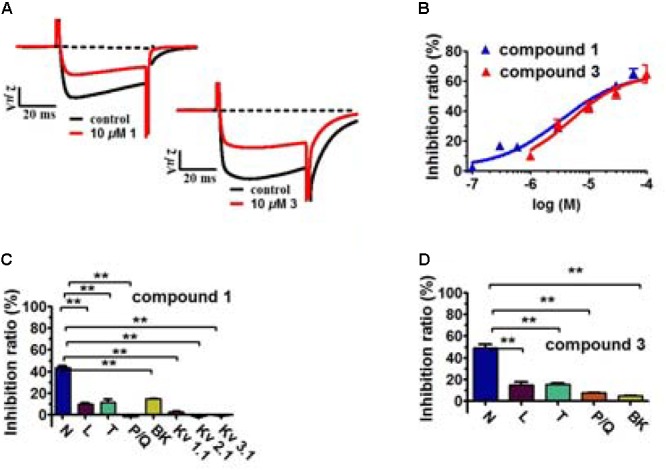
Inhibitory effects of compounds **1** and **3** on Ca_v_2.2 and their selectivity. **(A)** Representative Ca_v_2.2 current traces evoked by a 50-ms depolarization to +20 mV at 3-s intervals from a holding potential (HP) of –80 mV in the presence of the indicated compounds. Ca_v_2.2 was expressed in *Xenopus* oocytes. **(B)** Dose–response relationships of the indicated compounds for Ca_v_2.2 at a HP of –80 mV. Data points represent mean ± SEM of three or five measurements. Solid curve represents a fit to a modified Hill equation with an EC_50_ of 3.3 μM (**1**) and 4.8 μM (**3**), and a Hill coefficient of 0.82 (**1**) and 0.93 (**3**). **(C,D)** Effects of compounds **1** and **3** on indicated ion channels. The effect on the peak current was determined for each compound at 10 μM (N-, L-, P/Q- and T-type VGCCs) and 30 μM (BK, K_v_1.1, K_v_2.1, and K_v_3.1 potassium channels). Data represent mean ± SEM (*n* = 3). ^∗∗^*P* < 0.01 according to the two-tailed Student’s *t*-test.

The traditional use of the roots and rhizomes of *V. jatamansi* to treat abdominal pain led us to assume that some of their effects may be mediated through inhibition of Ca_v_2.2. Therefore, we carried out a systematic functional assay of the various extracts, fractions and isolated compounds obtained from the herbal medicine. Initially, we found that the ethanol extract and its EtOAc-soluble layer (0.01 mg/ml) showed inhibitory effects of 36 and 49% on Ca_v_2.2, respectively, while the *n*-BuOH and H_2_O layers (0.01 mg/ml) exhibited negligible activities (**Table 2**). Of all the fractions from the ethyl acetate extract, fractions 4 and 6 exhibited strongest activities on Ca_v_2.2, with 68.6 ± 3.1% and 60.1 ± 4.0% inhibition at 0.01 mg/ml, respectively (**Table 2**). Further phytochemical isolation from these two fractions led to the discovery of two new valepotriates, jatamanvaltrate T and U (**1**–**2**) and fifteen known compounds (**3**–**17**). All of the isolates were evaluated for their inhibitory effects on Ca_v_2.2. The new compound **1** (jatamanvaltrate T) and the main constituent **3** showed prominent inhibition of Ca_v_2.2 (**Table 2** and **Figure 3**). Other compounds produced no (**10**–**17**) or much weaker (**2, 4**–**9**) effects on Ca_v_2.2, even at a concentration of 30 μM (**Table 2**) (**Supplementary Figure S26**). Based on the bioassay results and the structure of these compounds, we postulate that the diene-type iridoid is a nucleus for the inhibitory activity on Ca_v_2.2.

**Table 2:** Effect of extracts and compounds 1-9 on Ca_v_2.2 N-type VGCC.

Fraction	Ca_v_2.2^a^ (N-type)	Compound	Ca_v_2.2^b^ (N-type)
EtOH extract	36.3 ± 10.2%	**1**	43.8 ± 1.7%
EtOAc-part	48.6 ± 7.7%	**2**	3.2 ± 2.1%
*n*-BuOH-part	–	**3**	38.9 ± 6.7%
H_2_O-part	1.1 ± 1.7%	**4**	4.1 ± 2.4%
**Fr.1**	1.3 ± 6.3%	**5**	9.0 ± 16.2%
**Fr.2**	56.5 ± 4.8%	**6**	8.5 ± 7.0%
**Fr.3**	10.5 ± 6.2%	**7**	0.1 ± 1.1%
**Fr.4**	68.6 ± 3.1%	**8**	3.4 ± 1.9%
**Fr.5**	55.2 ± 3.5%	**9**	5.4 ± 1.5%
**Fr.6**	60.1 ± 4.0%		
**Fr.7**	34.1 ± 6.3%		
**Fr.8**	28.4 ± 5.2%		
**Fr.9**	14.1 ± 2.0%		

*^*a*^Peak current inhibition ratio of extracts at 0.01 mg/ml; ^*b*^Peak current inhibition ratio of compounds at 10 μM (****1****and****3****) or 30 μM (other compounds); Data represent the mean ± SEM of 3 or 4 cells.*

The inhibition of Ca_v_2.2 by compounds **1** and **3** was dose-dependent, with an EC_50_ of 3.3 μM (*n* = 5) and 4.8 μM (*n* = 3), respectively (**Figure 3**). Interestingly, the inhibition by both compounds was incomplete, plateauing at ∼65% at a near saturation concentration of 100 μM (**Figure 3**). This result suggests that compounds **1** and **3** act allosterically to modulate Ca_v_2.2 gating rather than block channel conduction.

We also tested the effect of compounds **1** and **3** on several other types of VGCCs, including L-type (Ca_v_1.2), P/Q-type (Ca_v_2.1) and T-type (Ca_v_3.1), and on several types of potassium channels, including Kv1.1, Kv2.1, Kv3.1 and BK channels (**Table 3** and **Figures 3C,D**). At 10 μM, 1 and 3 showed substantially weaker inhibitory effects on Ca_v_1.2, Ca_v_2.1, and Ca_v_3.1 (**Table 3** and **Figures 3C,D**). At 30 μM, compound 1 had negligible effects on Kv1.1, Kv2.1, and Kv3.1 channels (**Table 3** and **Figure 3C**), although both compounds 1 and 3 showed weak inhibition on the BK channel (**Table 3** and **Figure 3D**).

**Table 3:** Effect of 1 and 3 on selected types of VGCCs and potassium channels.

Compound	Ca_v_1.2^a^ (L-type)	Ca_v_2.1^a^ (P/Q-type)	Ca_v_3.1^a^ (T-type)	BK^b^ channel	K_v_1.1^b^	K_v_2.1^b^	K_v_3.1^b^
1	10.1 ± 1.2%	1.3 ± 0.3%	11.8 ± 2.9%	15.5 ± 1.0%	2.8 ± 1.1%	1.0 ± 1.1%	0.5 ± 0.5%
3	13.5 ± 4.1%	7.8 ± 1.2%	20.4 ± 7.4%	6.7 ± 7.7%	–	–	–

*^*a*^Peak current inhibition ratio of compounds at 10 μM; ^*b*^Peak current inhibition ratio of compounds at 30 μM; “-” not tested, due to the limited amount of material. Data represent the mean ± SEM of 3 or 4 cells.*

In a positive control experiment, we tested the effect of two known N-type VGCC inhibitors, the divalent cation cadmium (Cd^2+^), which is a broad pore blocker of VGCCs, and the marine cone snail peptide toxin ω-conotoxin MVIIA, which is an N-type VGCC antagonist. As expected, both molecules inhibited Ca_v_2.2 currents in *Xenopus* oocytes and HEK 293T cells (**Supplementary Figures S27, S28**).

In summary, our study shows that valepotriates, one of the main constituents of the *V. jatamansi*, can inhibit Ca_v_2.2 N-type VGCCs. This activity is consistent with the analgesic effect of *V. jatamansi* in alleviating abdominal distention and pain. Further bioassay-guided search in *V. jatamansi* may yield more compounds that target N-type VGCCs.

## Author Contributions

F-WD and H-HJ are the co-first authors responsible for making experiments, consulting literature, and writing article. LY, YG, and C-TZ are responsible for editorial assistance with this manuscript. DY, C-JY, and HL are responsible for participating in plasma sample preparation. JZ took part in the topic selection and the experiment advising. JY, YN, and J-MH are corresponding authors of this article will be responsible for conducting the research and all correspondence with the editorial and accept the consultation of the reader.

## Conflict of Interest Statement

The authors declare that the research was conducted in the absence of any commercial or financial relationships that could be construed as a potential conflict of interest.
