# Supplementary material for: Valepotriates From the Roots and Rhizomes of Valeriana jatamansi Jones as Novel N-Type Calcium Channel Antagonists
Source: Front Pharmacol. 2018 Aug 13;9:885. doi: 10.3389/fphar.2018.00885 (PMC6099110; doi:10.3389/fphar.2018.00885)
Supplement: Supplementary file 1 [file Data_Sheet_1.pdf]

Supplementary Material

Valepotriates from the roots and rhizomes of *Valeriana jatamansi* Jones as novel N-type Calcium channel antagonists

Fa-Wu Dong[1,2,3†], He-Hai Jiang[4,5,6,7†], Liu Yang[1,2], Ye Gong[4,5,6], Cheng-Ting Zi[1,2], Dan Yang[1,2], Chen-Jun Ye[4,5,6], Huan Li[4,5,6], Jian Yang[4,5,6,8*], Yin Nian[4,5,6*], Jun Zhou[1,2*] and Jiang-Miao Hu[1,2*]

[†] these authors contribute equally

*** Correspondence:**

E-mail: jianyang@mail.kiz.ac.cn (J. Yang), nianyin@mail.kiz.ac.cn (Y. Nian), jzhou@mail.kib.ac.cn or hujiangmiao@mail.kib.ac.cn (J.-M. Hu).

FIGURE S1. HRESIMS of jatamanvaltrate T (**1**). S3
FIGURE S2. ^{1}H NMR (Bruker AV-600, 600 MHz, DMSO) of jatamanvaltrate T (**1**)...................S3
FIGURE S3. ^{1}H NMR (Bruker AM-400, 400 MHz, CD_3OD) of jatamanvaltrate T (**1**). S4
FIGURE S4. ^{13}C NMR (Bruker AM-400, 100 MHz, CD_3OD) of jatamanvaltrate T (**1**). S4
FIGURE S5. HSQC (Bruker DRX-500, 500 MHz, CD_3OD) of jatamanvaltrate T (**1**). S5
FIGURE S6. HMBC (Bruker DRX-500, 500 MHz, CD_3OD) of jatamanvaltrate T (**1**). S5
FIGURE S7. ^{1}H-^{1}H COSY (Bruker DRX-500, 500 MHz, CD_3OD) of jatamanvaltrate T (**1**).........S6
FIGURE S8. ROESY (Bruker AV-600, 600 MHz, DMSO) of jatamanvaltrate T (**1**)..................S6
FIGURE S9. ROESY (Bruker DRX-500, 500 MHz, CD_3OD) of jatamanvaltrate T (**1**)..................S7
FIGURE S10. IR spectrum of jatamanvaltrate T (**1**)..S7
FIGURE S11. UV spectra of jatamanvaltrate T (**1**)..S8
FIGURE S12. $[\alpha]_D$ spectra of jatamanvaltrate T (**1**)..S8
FIGURE S13. HRESIMS of jatamanvaltrate U (**2**)..S9
FIGURE S14. ^{1}H NMR (Bruker AM-400, 400 MHz, CD_3OD) of jatamanvaltrate U (**2**)..................S9
FIGURE S15. ^{13}C NMR (Bruker AM-400, 100 MHz, CD_3OD) of jatamanvaltrate U (**2**)............S10
FIGURE S16. HSQC (Bruker DRX-500, 500 MHz, CD_3OD) of jatamanvaltrate U (**2**)...............S10
FIGURE S17. HMBC (Bruker DRX-500, 500 MHz, CD_3OD) of jatamanvaltrate U (**2**)....... ....S11
FIGURE S18. ^{1}H-^{1}H COSY (Bruker DRX-500, 500 MHz, CD_3OD) of jatamanvaltrate U (**2**).......S11

FIGURE S19. ROESY (Bruker DRX-500, 500 MHz, CD_3OD) of jatamanvaltrate U (**2**)…….......…S12

FIGURE S20. IR spectrum of jatamanvaltrate U (**2**)..S12

FIGURE S21. UV spectra of jatamanvaltrate U (**2**)…………………………………………………S13

FIGURE S22. $[\alpha]_D$ spectra of jatamanvaltrate U (**2**)………………………………………………S13

FIGURE S23. | Effect of the fractions of the ethanol extract, EtOAc, n-BuOH and H_2O layers on Ca_v 2.2 expressed in *Xenopus* oocytes, n=3. A-D. Normalized current-voltage (I-V) curves of Ca_v2.2 in the absence and presence of 0.01 mg/ml……………………… ….S14

FIGURE S24. The inhibitory effects of the fractions of EtOAc layer on N type calcium channels ..S15

FIGURE S25. The inhibitory effects of **1**and **3** on L type, T type, P/Q type, BK type, Kv 1.2 type, Kv 2.1 type and Kv 3.1 type calcium channels……………………………………..….S16

FIGURE S26. The inhibitory effects of jatamanvaltrate U (**2**), volvaltrate B (**4**), 8,11-desoidodidrovaltrate (**5**), baldrinal (**6**), homobaldrinal (**7**), desacylbaldrinal (**8**) and 11-methoxyviburtinal (**9**) on N type calcium channels…………………………………S17

FIGURE S27. Inhibition of N type calcium channel expressed in *Xenopus* oocytes and HEK 293T cells by cadmium………………………………………………………………… S18

FIGURE S28. Inhibition of N type calcium channel expressed in *Xenopus* oocytes and HEK 293T cells by ω-conotoxin MVIIA…………………………………………………………S19

User Spectra

Fragmentor Voltage	Collision Energy	Ionization Mode
200	0	ESI

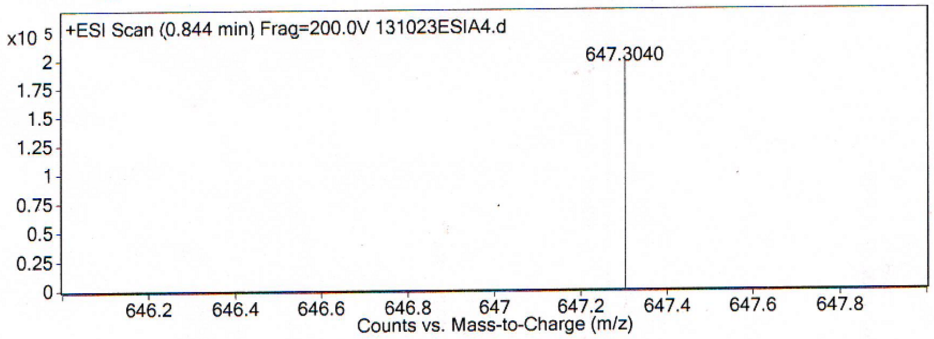

Formula Calculator Element Limits

Element	Min	Max
C	0	200
H	0	400
O	11	13
Na	1	1

Formula Calculator Results

Formula	Calculated Mass	Measured Mass	Diff. (mDa)	Diff. (ppm)	DBE
C32 H48 Na O12	647.3043	647.3045	-0.2	-0.3	8.5

FIGURE S1 | HRESIMS of jatamanvaltrate T (**1**).

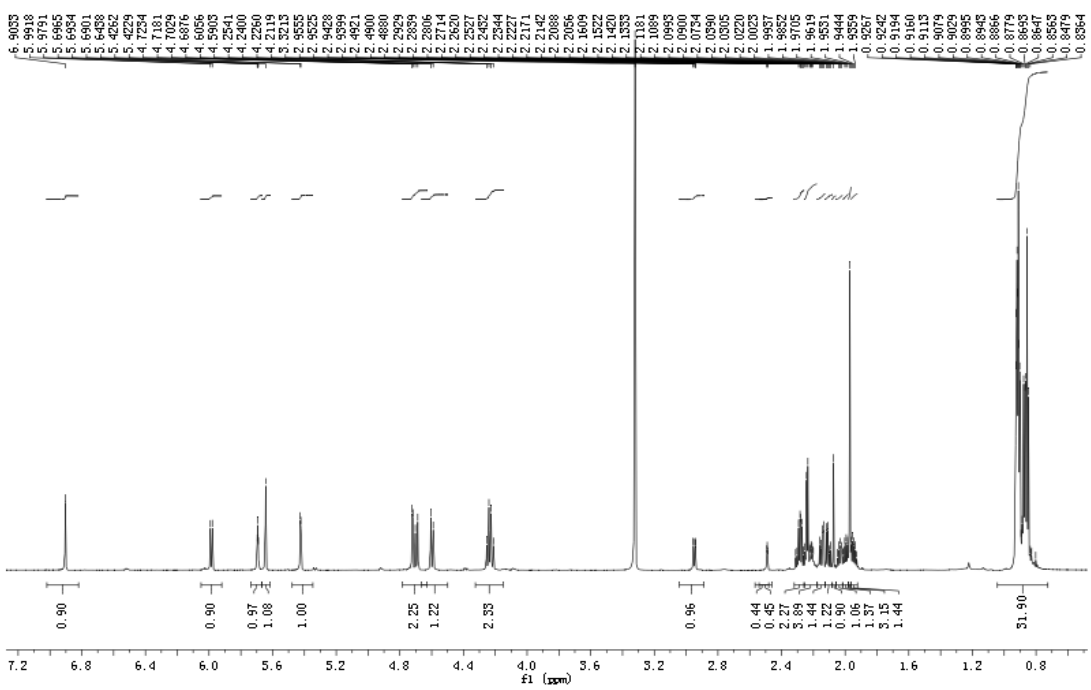

FIGURE S2 | ^{1}H NMR (Bruker AV-600, 600 MHz, DMSO) of jatamanvaltrate T (**1**).

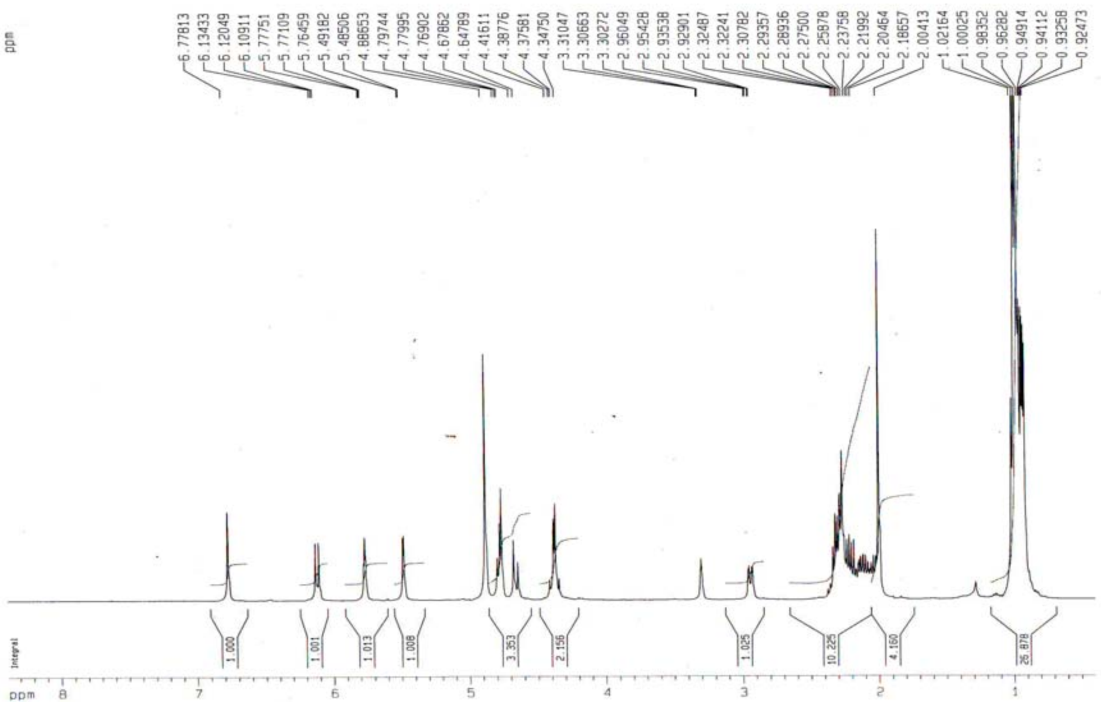

FIGURE S3 | ^{1}H NMR (Bruker AM-400, 400 MHz, CD_3OD) of jatamanvaltrate T (**1**).

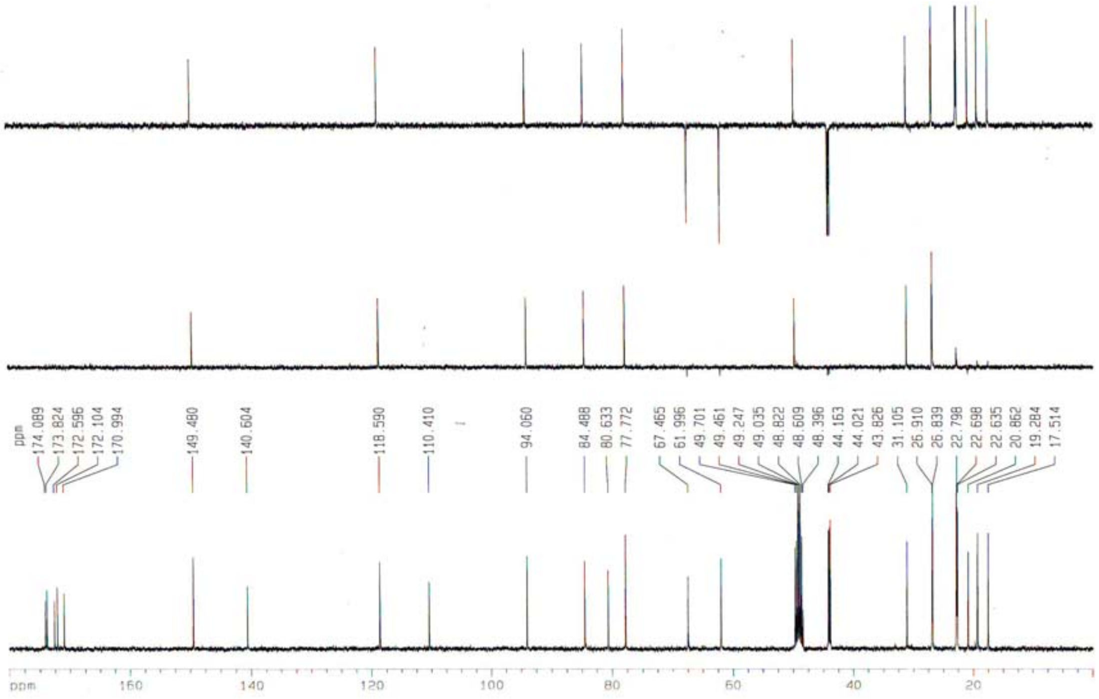

FIGURE S4 | ^{13}C NMR (Bruker AM-400, 100 MHz, CD_3OD) of jatamanvaltrate T (**1**).

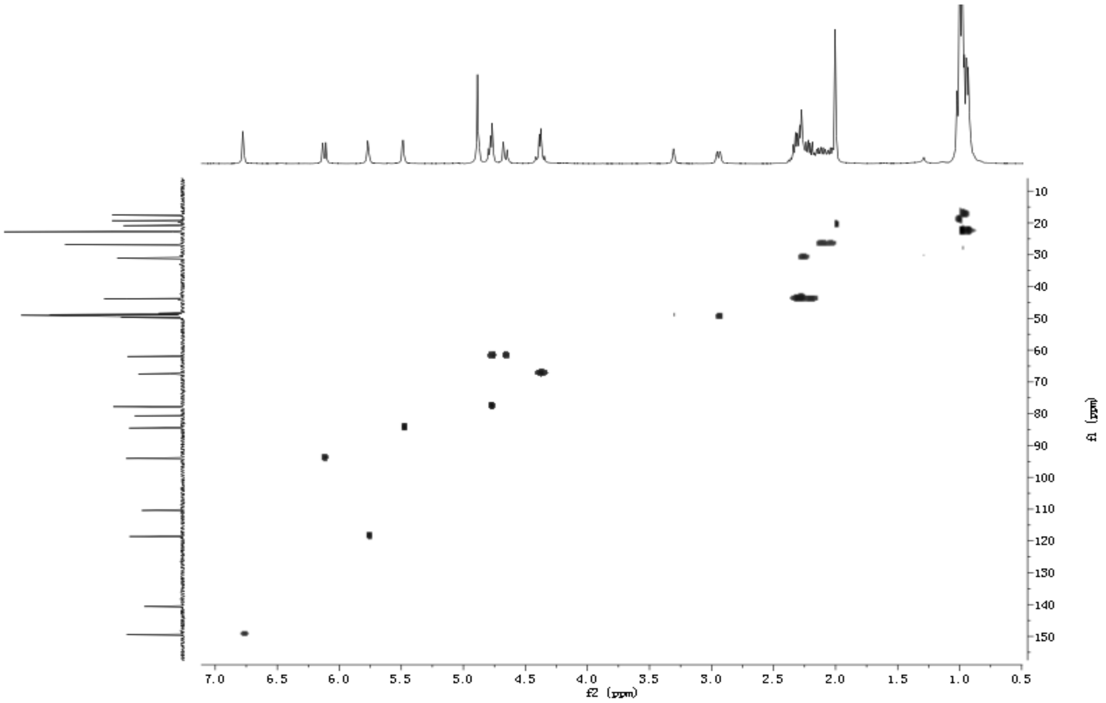

FIGURE S5 | HSQC (Bruker DRX-500, 500 MHz, CD_3OD) of jatamanvaltrate T (**1**).

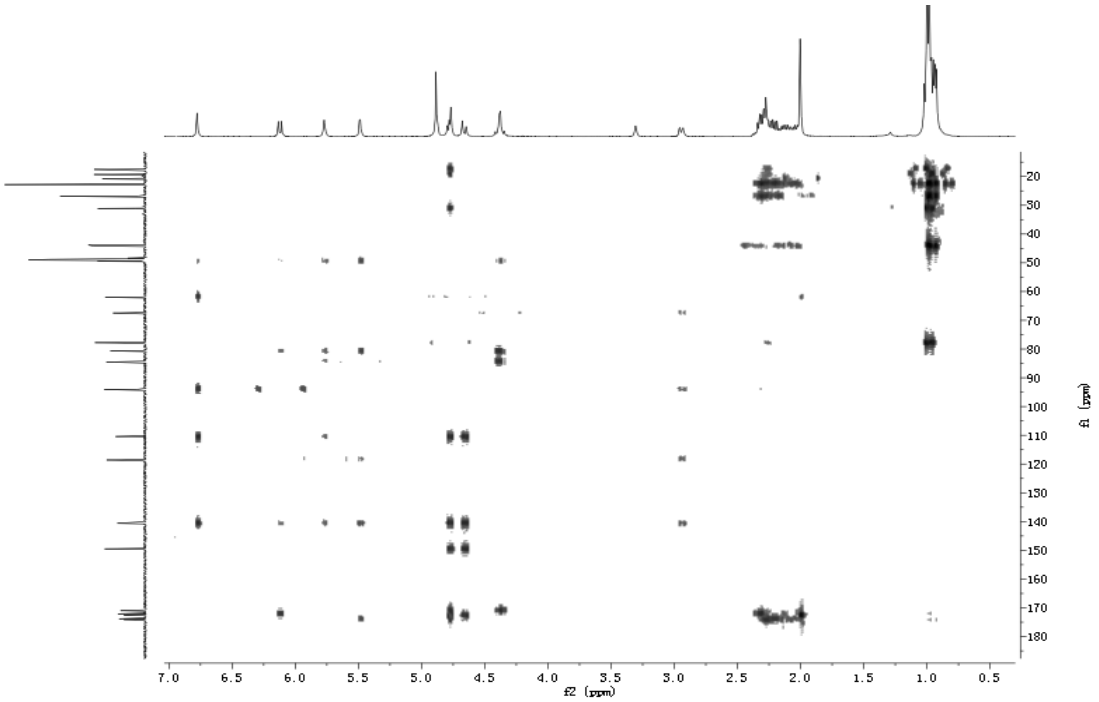

FIGURE S6 | HMBC (Bruker DRX-500, 500 MHz, CD_3OD) of jatamanvaltrate T (**1**).

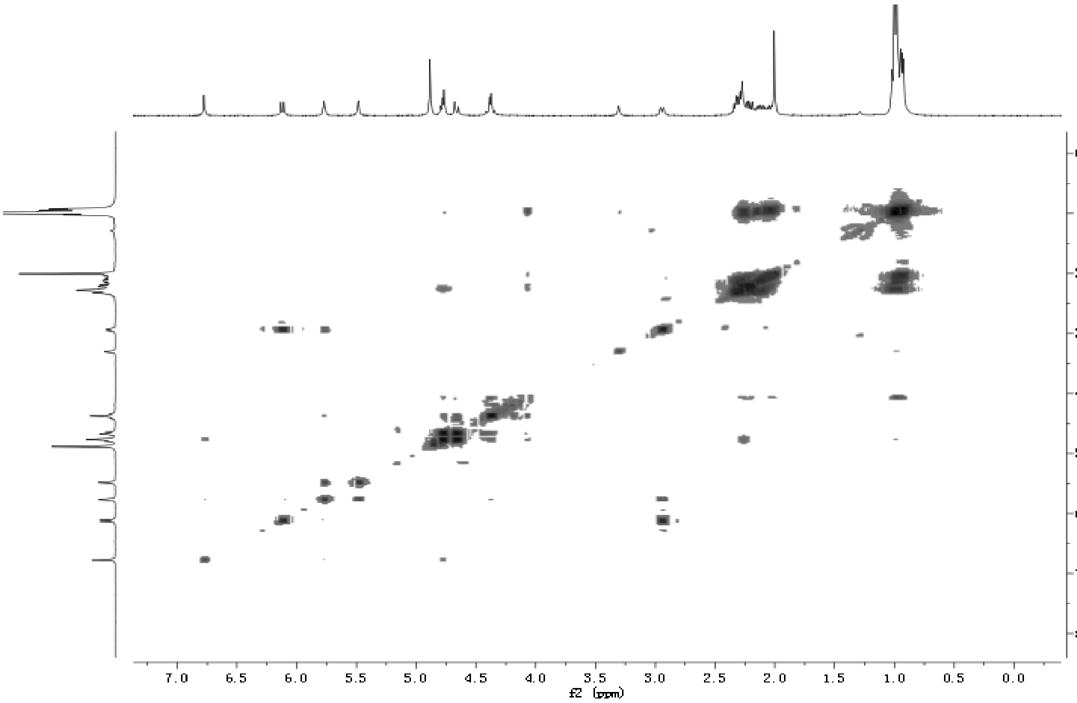

FIGURE S7 | ^{1}H-^{1}H COSY (Bruker DRX-500, 500 MHz, CD_3OD) of jatamanvaltrate T (**1**).

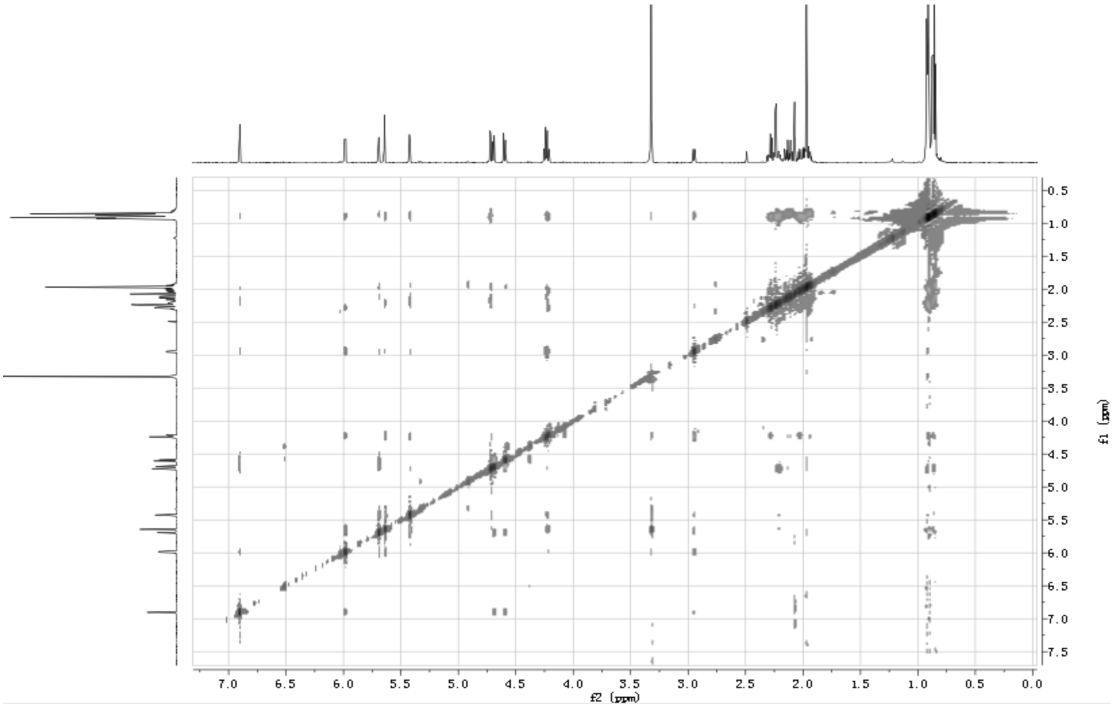

FIGURE S8 | ROESY (Bruker AV-600, 600 MHz, DMSO) of jatamanvaltrate T (**1**).

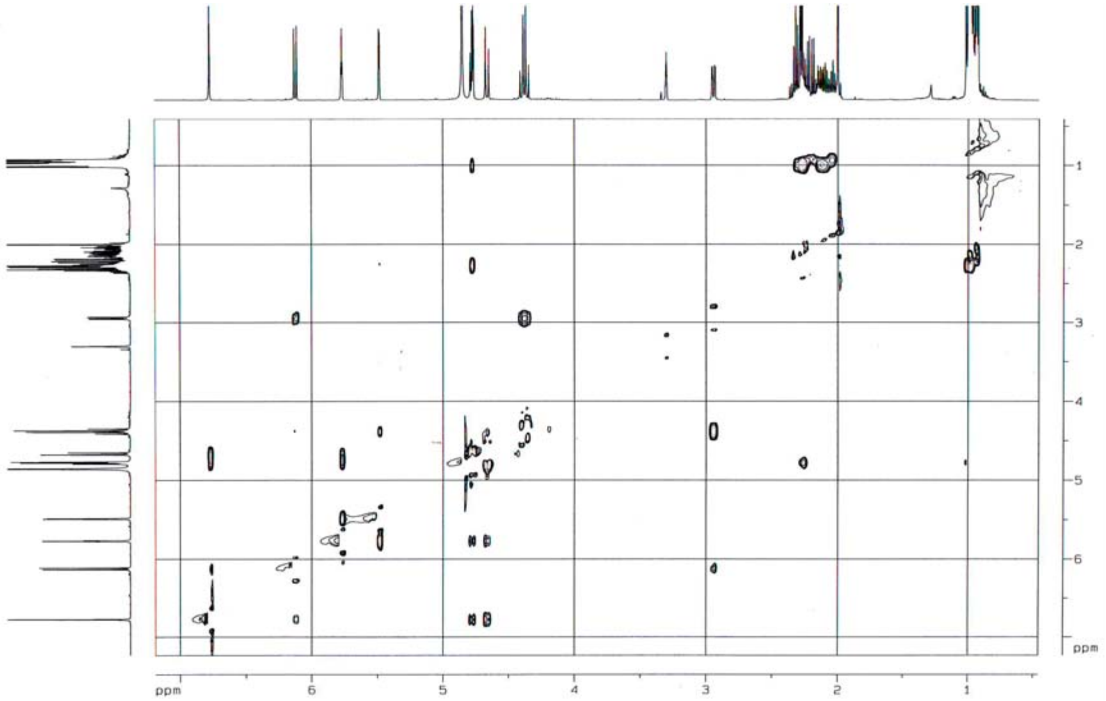

FIGURE S9 | ROESY (Bruker DRX-500, 500 MHz, CD_3OD) of jatamanvaltrate T (**1**).

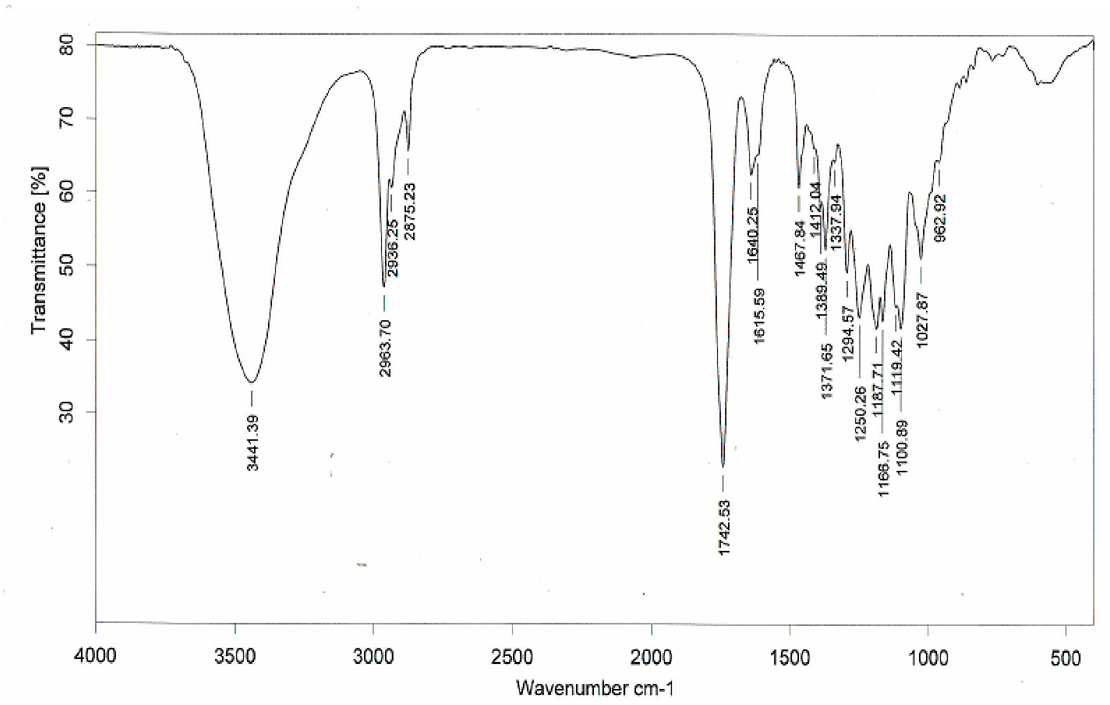

FIGURE S10 | IR spectrum of jatamanvaltrate T (**1**).

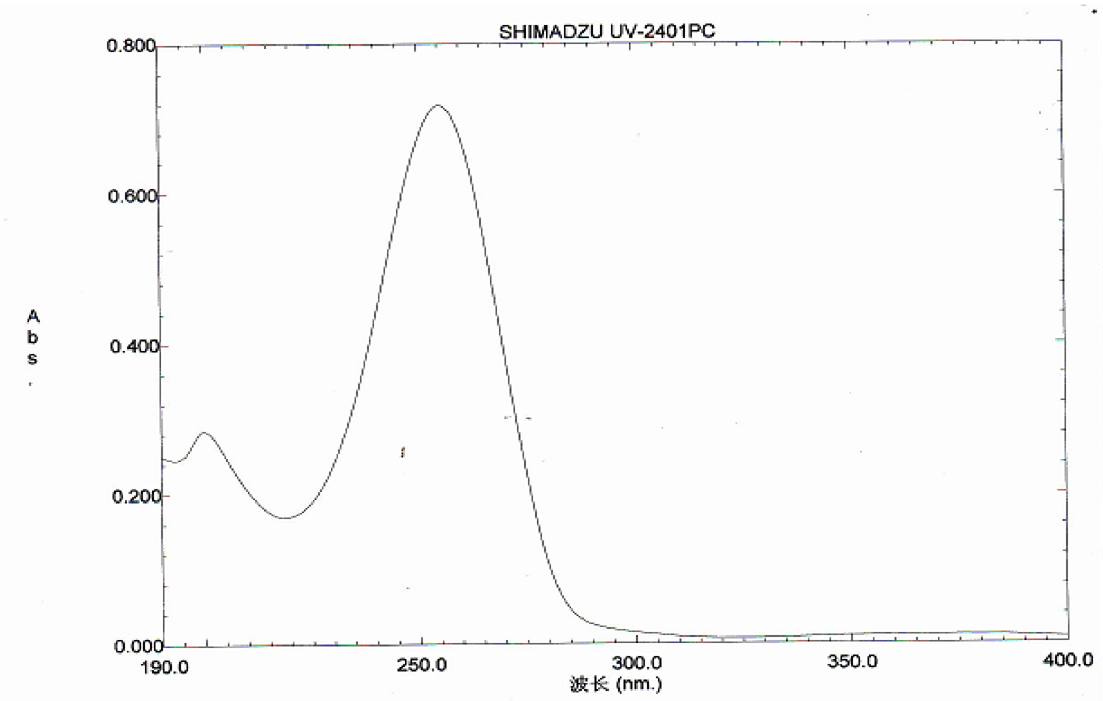

FIGURE S11 | UV spectrum of jatamanvaltrate T (**1**).

Optical rotation measurement

Model : P-1020 (A060460638)

No.	Sample	Mode	Data	Monitor / Blank	Temp. / Cell / Temp Point	Date / Comment / Sample Name	Light / Filter / Operator	Cycle Time / Integ Time
No.1	12 (1/3)	Sp.Rot	152.3480	0.2011	19.5	Wed Dec 10 18:32:57 2014	Na	2 sec
				0.0000	50.00	0.00264mg/mL MeOH	589nm	10 sec
					Cell	ZJV-27		
No.2	12 (2/3)	Sp.Rot	152.0450	0.2007	19.5	Wed Dec 10 18:33:10 2014	Na	2 sec
				0.0000	50.00	0.00264mg/mL MeOH	589nm	10 sec
					Cell	ZJV-27		
No.3	12 (3/3)	Sp.Rot	153.4090	0.2025	19.6	Wed Dec 10 18:33:23 2014	Na	2 sec
				0.0000	50.00	0.00264mg/mL MeOH	589nm	10 sec
					Cell	ZJV-27		

+152.6010°

FIGURE S12 | $[\alpha]_D$ spectrum of jatamanvaltrateT (**1**).

User Spectra

Fragmentor Voltage	Collision Energy	Ionization Mode
200	0	ESI

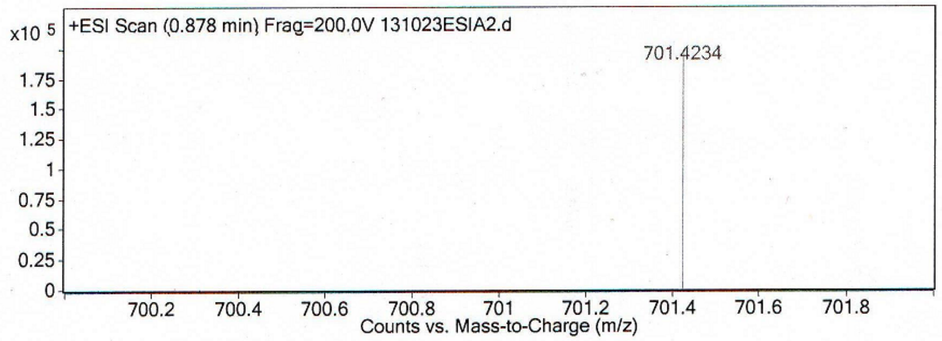

Formula Calculator Element Limits

Element	Min	Max
C	0	200
H	0	400
O	9	11
Na	1	1

Formula Calculator Results

Formula	Calculated Mass	Measured Mass	Diff. (mDa)	Diff. (ppm)	DBE
C38 H62 Na O10	701.4241	701.4239	0.1	0.2	7.5

FIGURE S13 | HRESIMS of jatamanvaltrate U (**2**).

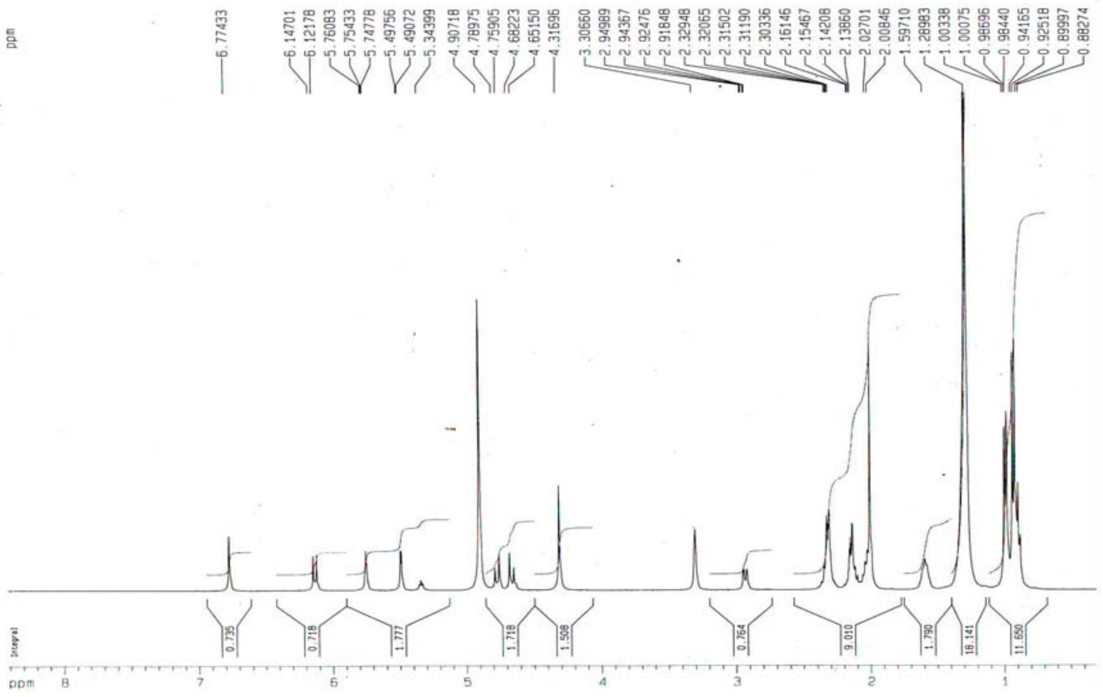

FIGURE S14 | ^{1}H NMR (Bruker AM-400, 400 MHz, CD_3OD) of jatamanvaltrate U (**2**).

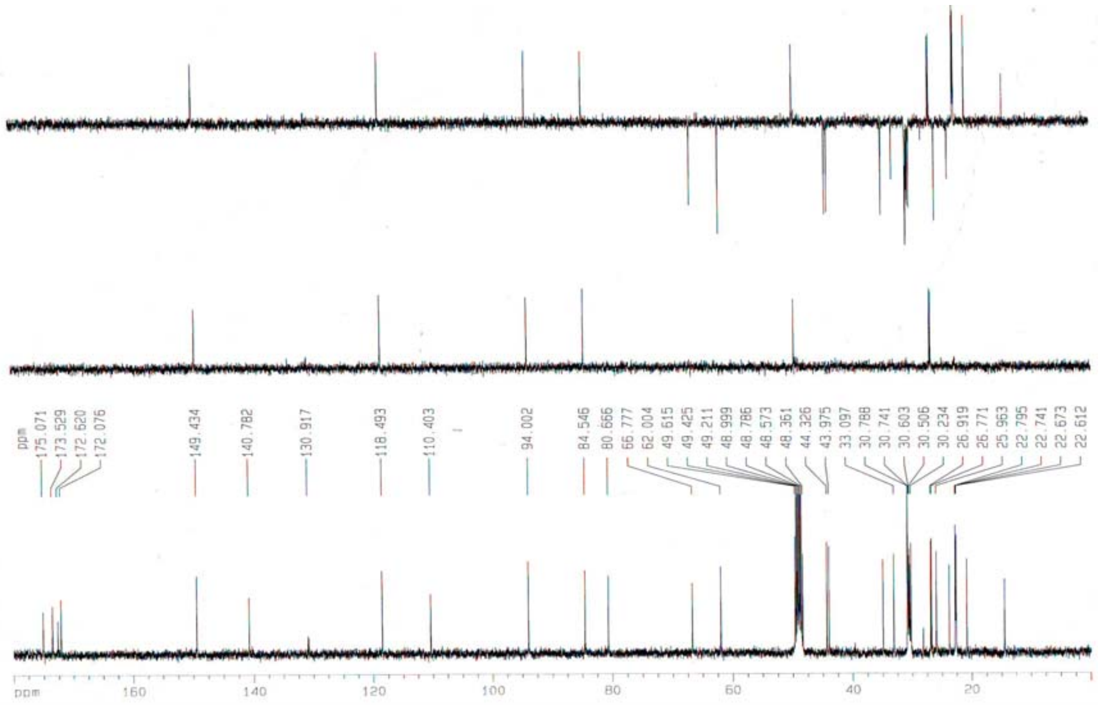

FIGURE S15 | ^{13}C NMR (Bruker AM-400, 100 MHz, CD_3OD) of jatamanvaltrate U (**2**).

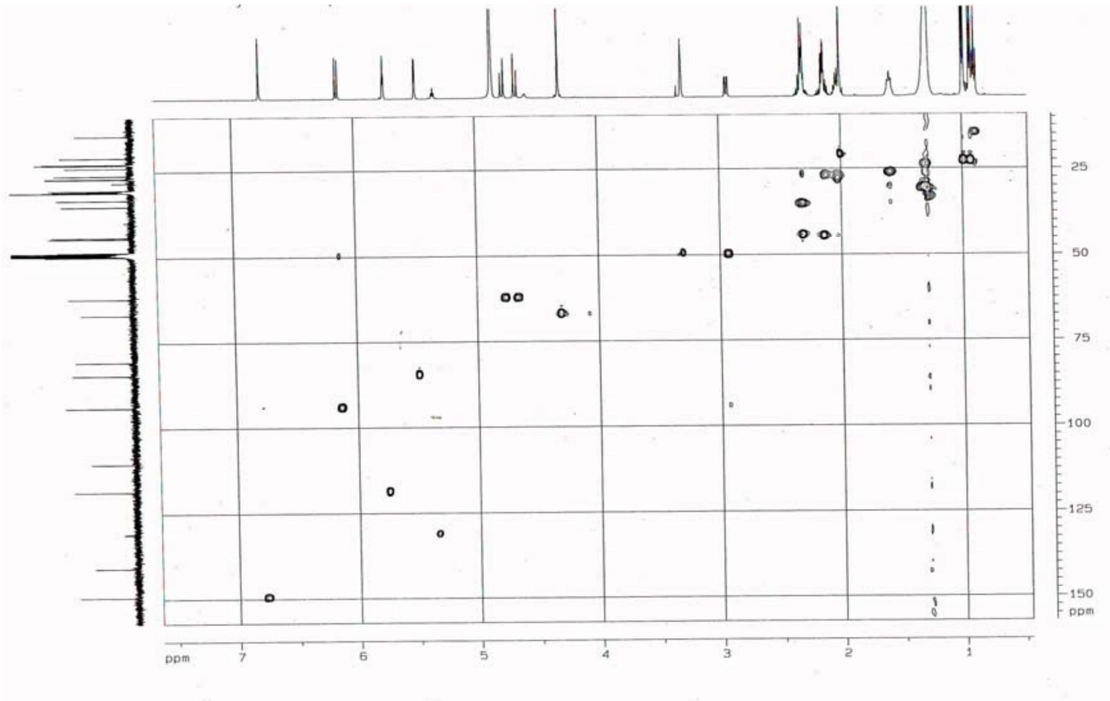

FIGURE S16 | HSQC (Bruker DRX-500, 500 MHz, CD_3OD) of jatamanvaltrate U (**2**).

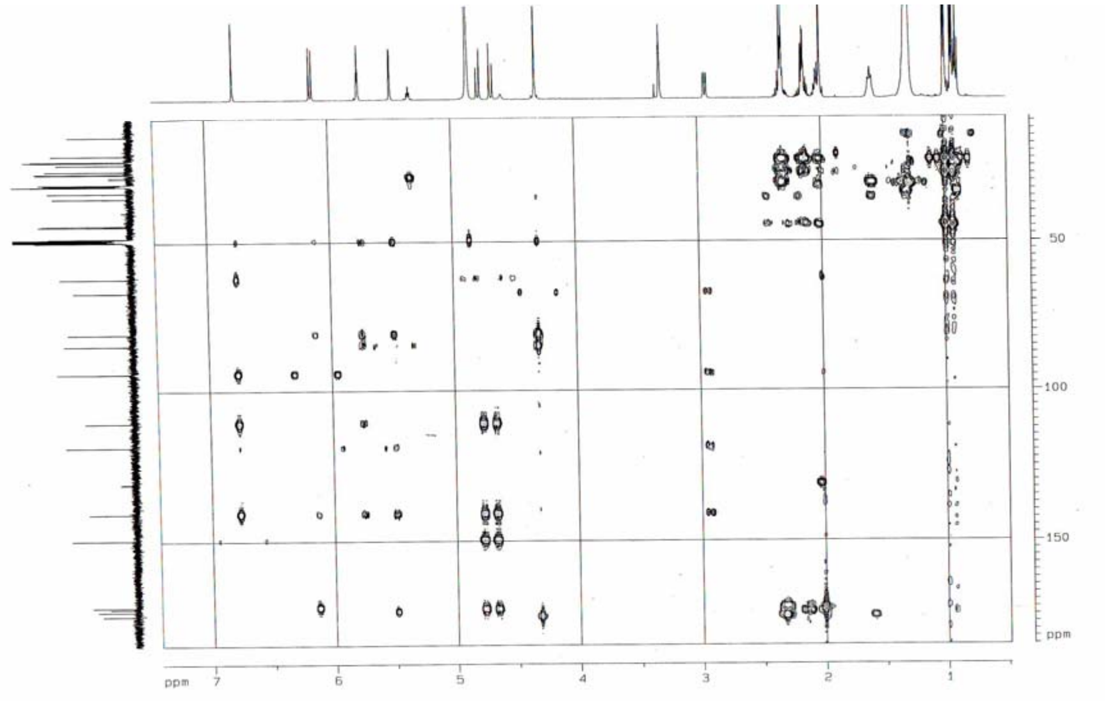

FIGURE S17 | HMBC (Bruker DRX-500, 500 MHz, CD_3OD) of jatamanvaltrate U (**2**).

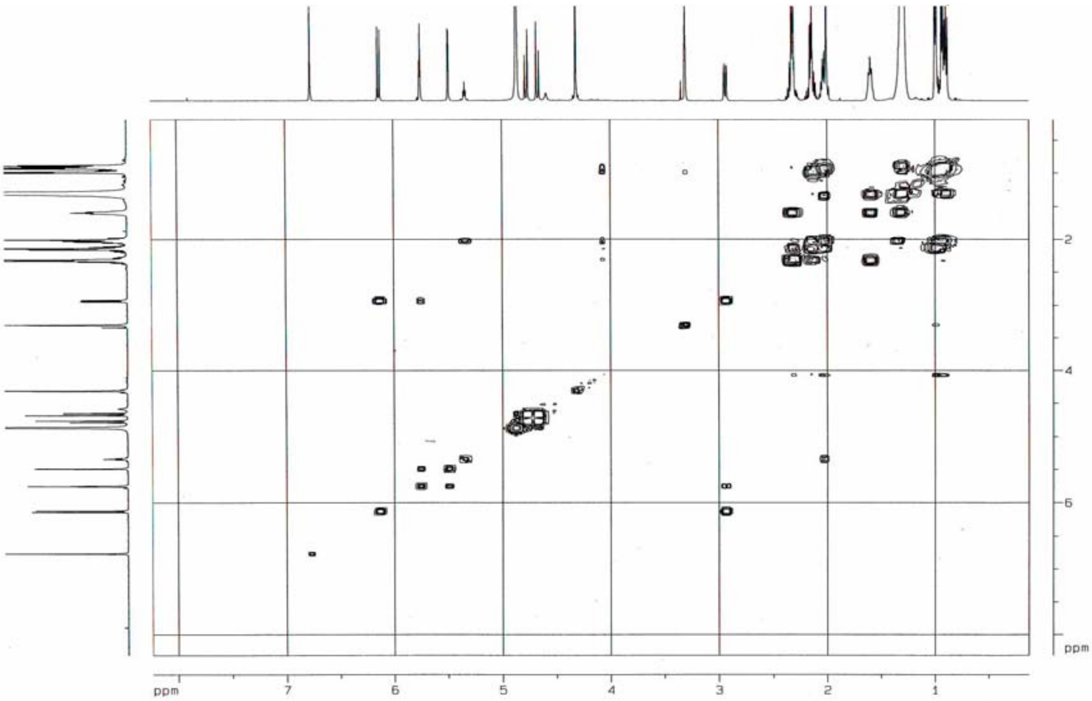

FIGURE S18 | ^1H-^1H COSY (Bruker DRX-500, 500 MHz, CD_3OD) of jatamanvaltrate U (**2**).

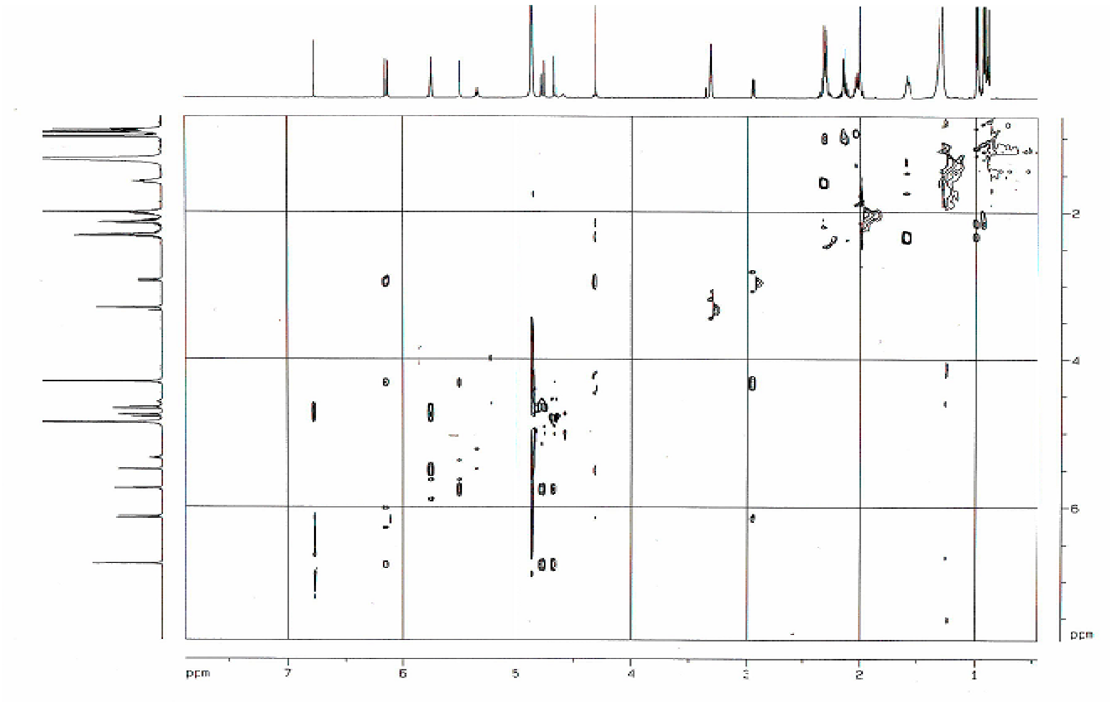

FIGURE S19 | ROESY (Bruker DRX-500, 500 MHz, CD_3OD) of jatamanvaltrate U (**2**).

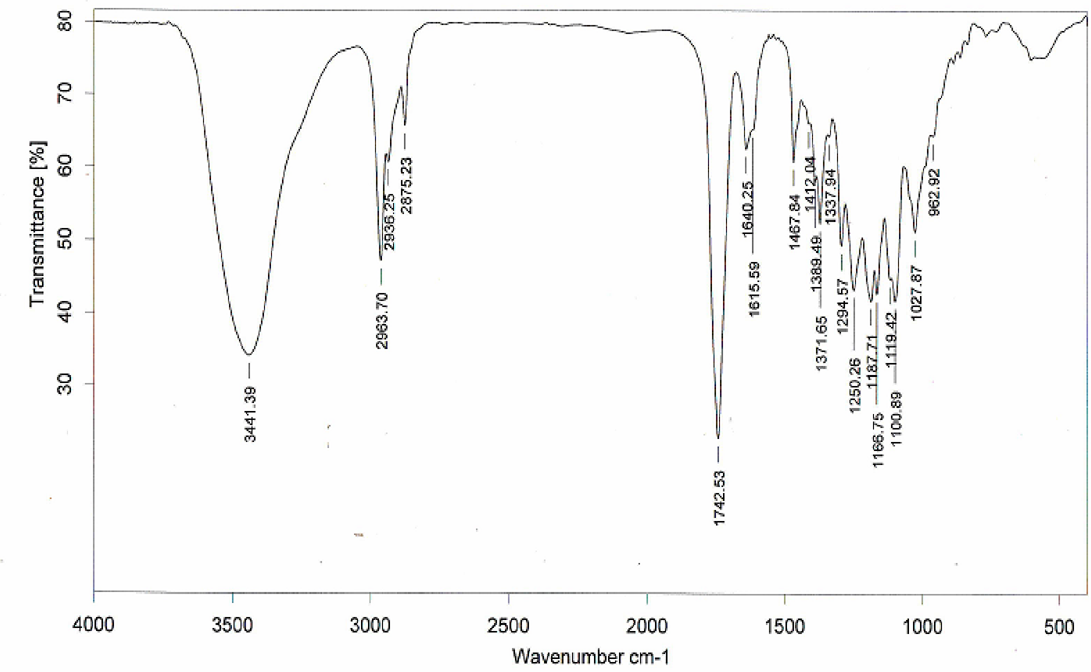

FIGURE S20 | IR spectrum of jatamanvaltrate U (**2**).

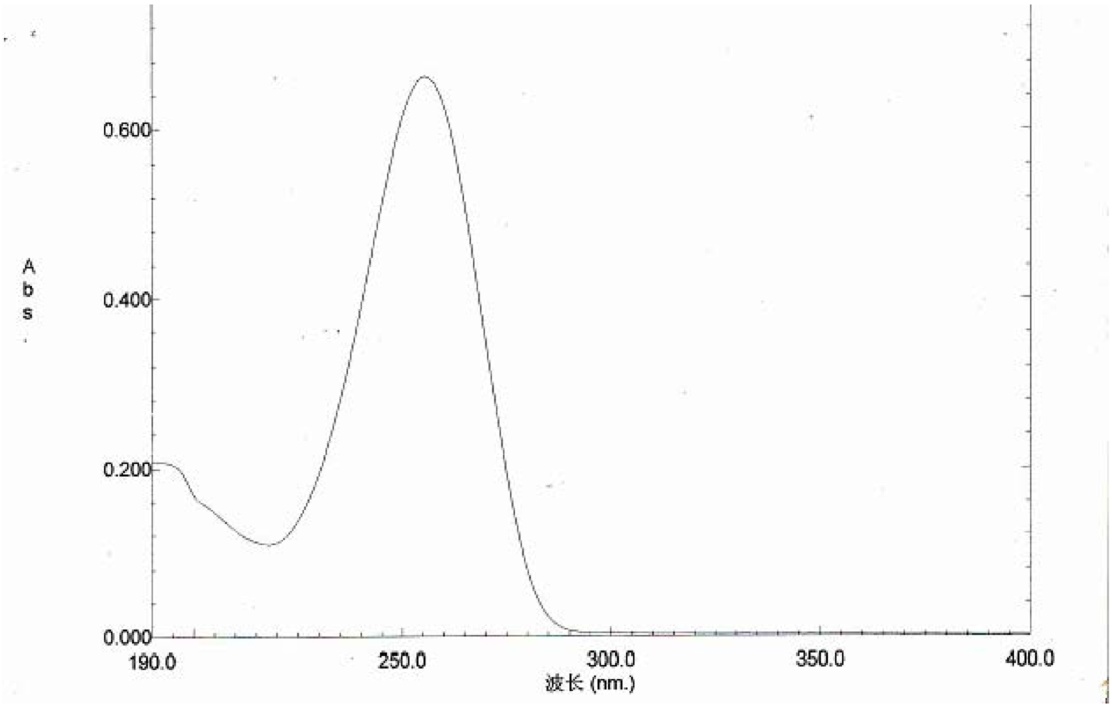

FIGURE S21 | UV spectrum of jatamanvaltrate U (**2**).

Optical rotation measurement

Model : P-1020 (A060460638)

No.	Sample	Mode	Data	Monitor<br>Blank	Temp.<br>Cell<br>Temp Point	Date<br>Comment<br>Sample Name	Light<br>Filter<br>Operator	Cycle Time<br>Integ Time
No.1	6 (1/3)	Sp.Rot	131.2470	0.2999<br>0.0000	17.0<br>50.00<br>Cell	Wed Oct 23 14:51:08 2013<br>0.00457g/ml MeOH<br>ZJV-25	Na<br>589nm	2 sec<br>10 sec
No.2	6 (2/3)	Sp.Rot	131.8160	0.3012<br>0.0000	17.0<br>50.00<br>Cell	Wed Oct 23 14:51:21 2013<br>0.00457g/ml MeOH<br>ZJV-25	Na<br>589nm	2 sec<br>10 sec
No.3	6 (3/3)	Sp.Rot	131.7720	0.3011<br>0.0000	17.0<br>50.00<br>Cell	Wed Oct 23 14:51:35 2013<br>0.00457g/ml MeOH<br>ZJV-25	Na<br>589nm	2 sec<br>10 sec

+131.6120

FIGURE S22 | $[\alpha]_D$ spectrum of jatamanvaltrateU (**2**).

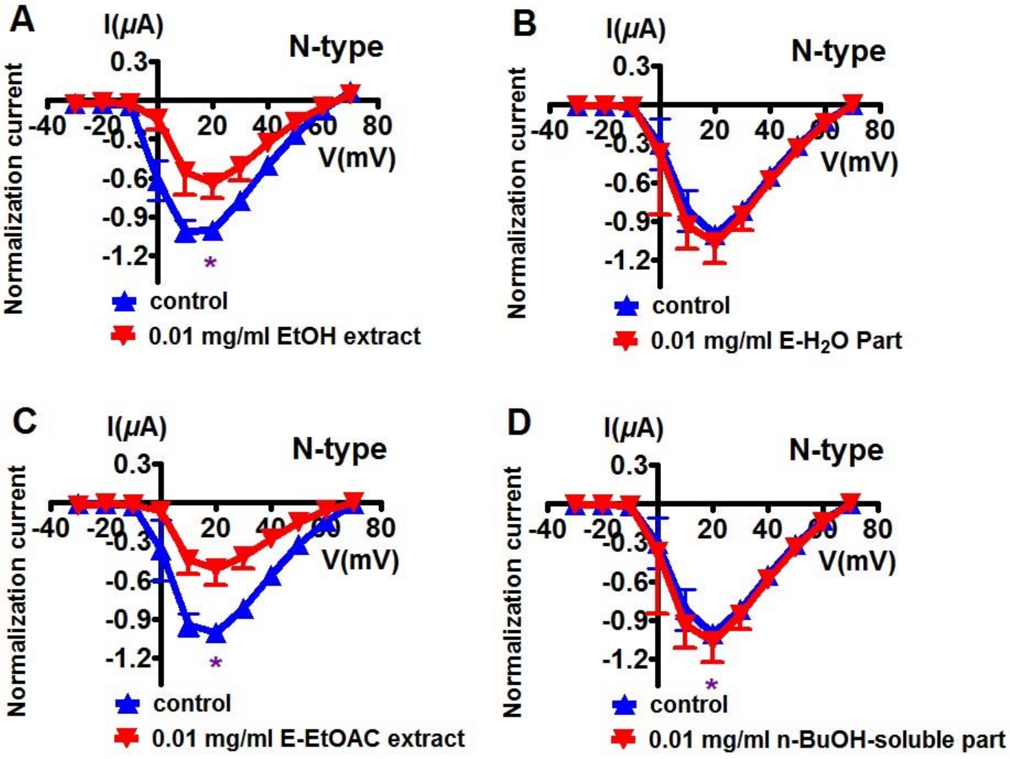

FIGURE S23 | Effect of the fractions of the ethanol extract, EtOAc, n-BuOH and H_2O layers on Ca_v 2.2 expressed in *Xenopus* oocytes, n=3. A-D. Normalized current-voltage (I-V) curves of $Ca_v2.2$ in the absence and presence of 0.01 mg/ml.

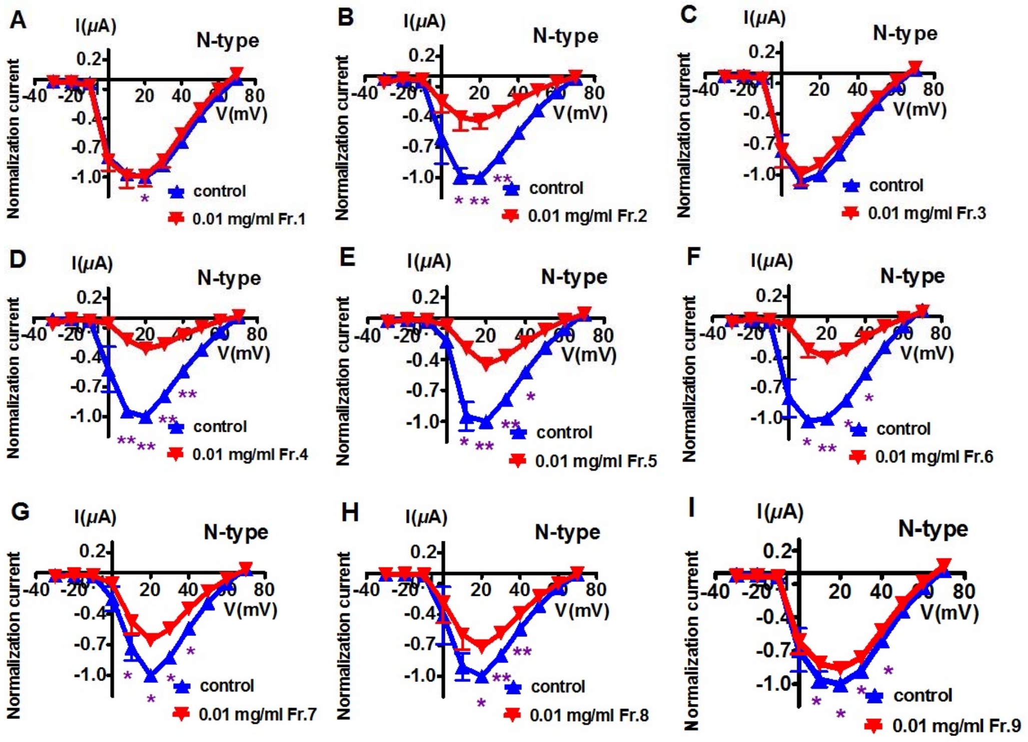

FIGURE S24 | Effect of the fractions of EtOAc layer on Ca_v 2.2 expressed in *Xenopus* oocytes, n=3. A-I. Normalized current-voltage (I-V) curves of $Ca_v2.2$ in the absence and presence of 0.01 mg/ml Fr1-Fr9.

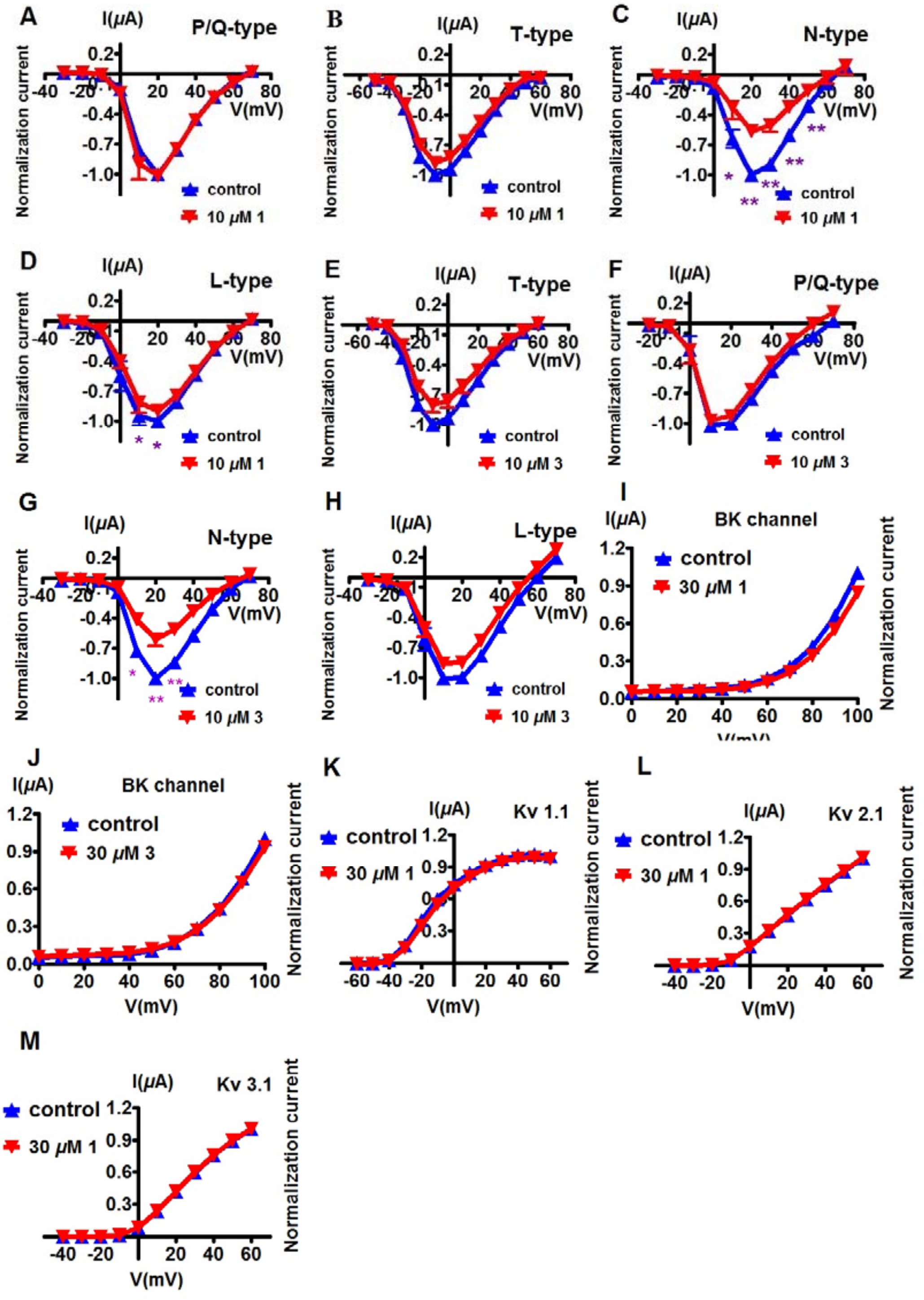

FIGURE S25 | Effect of jatamanvaltrate T (**1**) and valtrate hydrine B8 (**3**) on voltage-gated calcium channels (P/Q type, T type, N type and L type), BK channel, Kv 1.1, Kv 2.1 and Kv 3.1 expressed in *Xenopus* oocytes. Normalized

current-voltage (I-V) curves of the indicated channels are shown in the absence and presence of the indicated compounds.

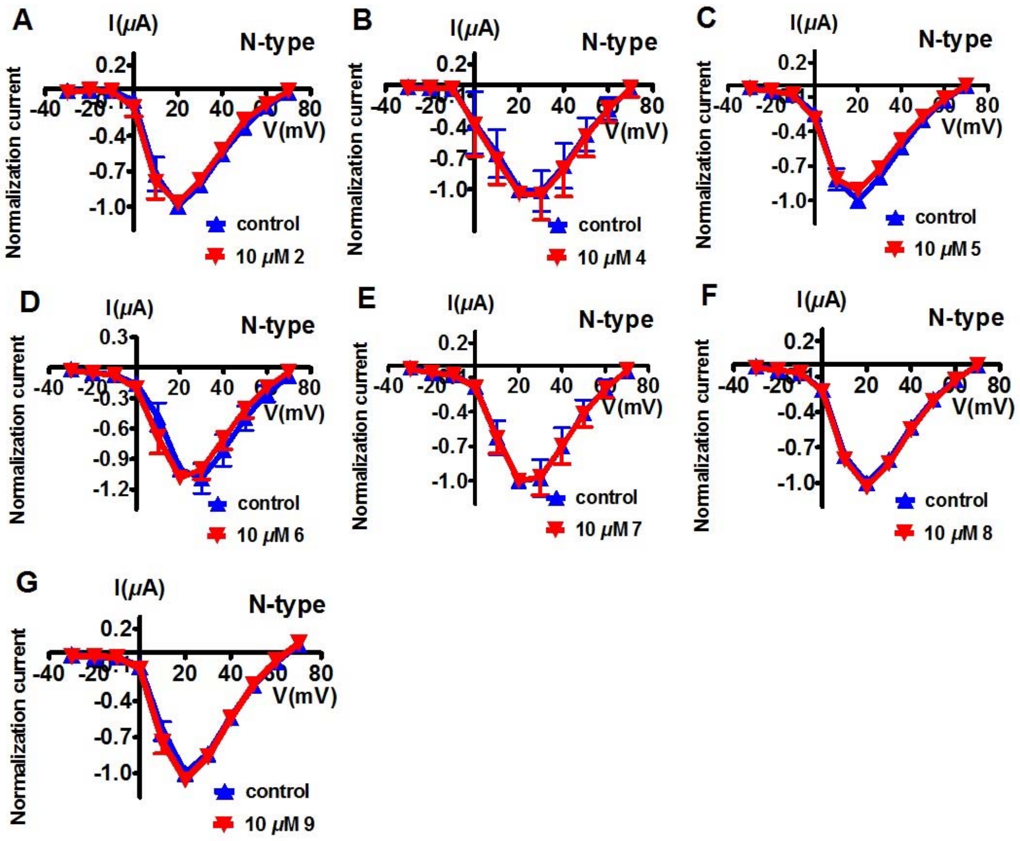

FIGURE S26 | Effect of jatamanvaltrate U (**2**), volvaltrate B (**4**), 8,11-desoidodidrovaltrate (**5**), baldrinal (**6**), homobaldrinal (**7**), desacylbaldrinal (**8**) and 11-methoxyviburtinal (**9**) on N type calcium channels expressed in *Xenopus* oocytes. A-G: Normalized current-voltage (I-V) curves of $Ca_v2.2$ in the absence and presence of the indicated compounds.

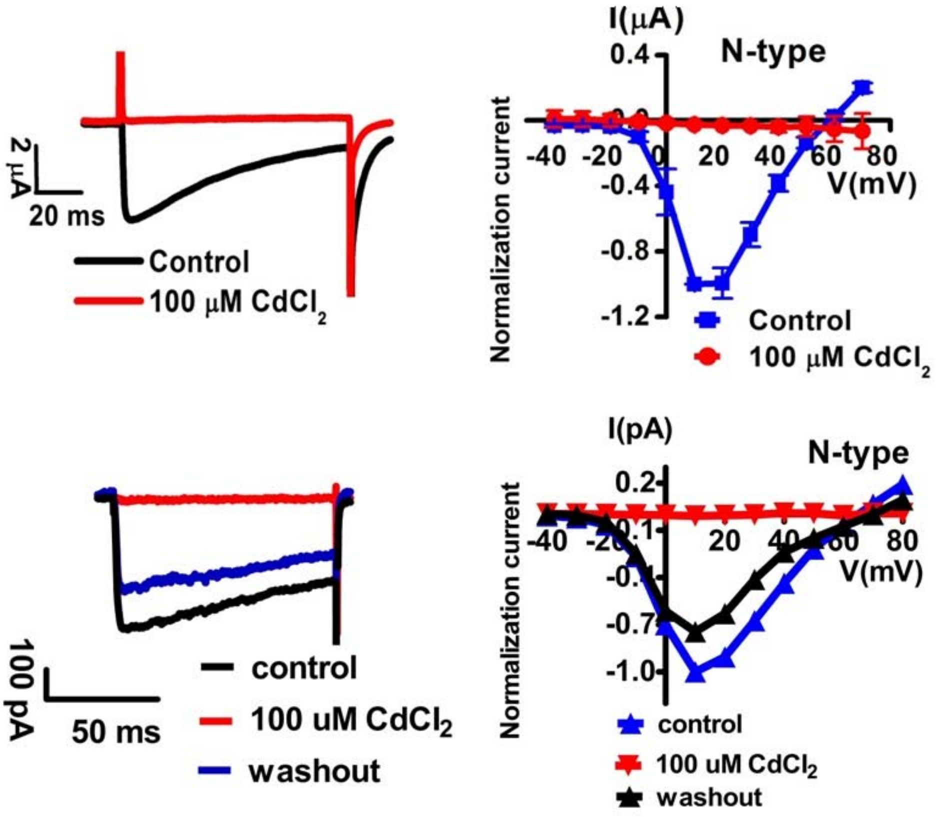

FIGURE S27 | Inhibition of N type calcium channel expressed in *Xenopus* oocytes and HEK 293T cells by cadmium. Top panels: TEVC recordings from *Xenopus* oocytes. Lower panels: Whole-cell recordings from HEK 293T cells. Left panels: Current traces recorded at + 10 mV before, during and after bath application of 100 μM $CdCl_2$. Similar results were obtained in 3-5 cells. Right panels: Normalized current-voltage (I-V) curves of $Ca_v2.2$ before, during and after bath application of 100 μM $CdCl_2$.

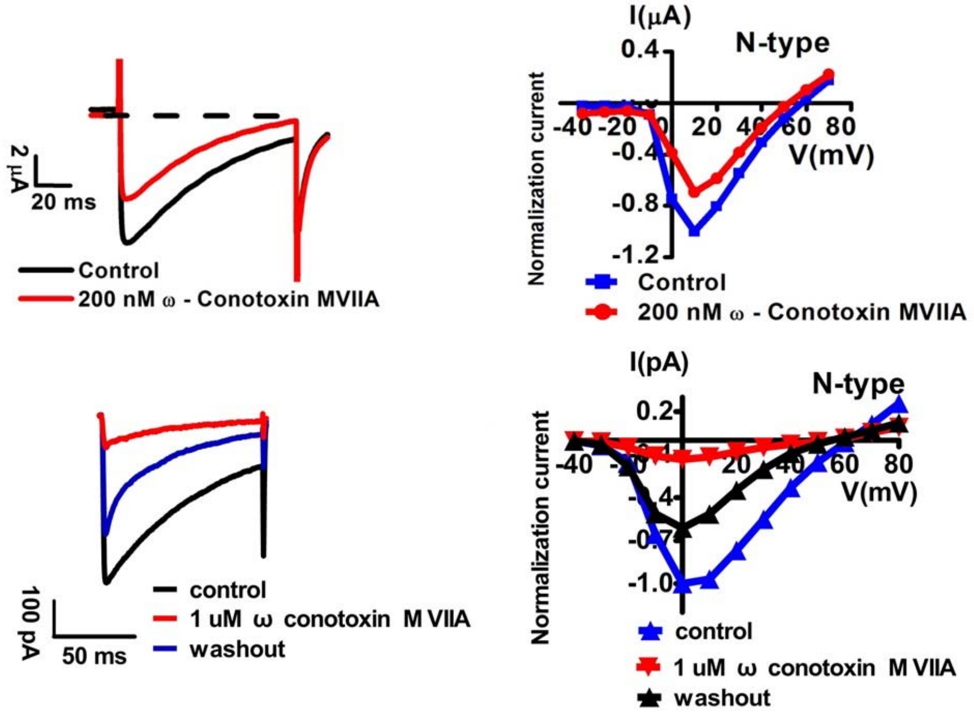

FIGURE S28 | Inhibition of N type calcium channel expressed in *Xenopus* oocytes and HEK 293T cells by ω-conotoxin MVIIA. Top panels: TEVC recordings from *Xenopus* oocytes. Lower panels: Whole-cell recordings from HEK 293T cells. Left panels: Current traces recorded at + 10 mV before, during and after bath application of the indicated concentrations of ω-conotoxin MVIIA. Similar results were obtained in 3-5 cells. Right panels: Normalized current-voltage (I-V) curves of $Ca_v2.2$ before, during and after bath application of the indicated concent
